# Bimetallic Mesoporous MCM-41 Nanoparticles with Ta/(Ti, V, Co, Nb) with Catalytic and Photocatalytic Properties

**DOI:** 10.3390/nano14242025

**Published:** 2024-12-16

**Authors:** Viorica Parvulescu, Gabriela Petcu, Nicoleta G. Apostol, Irina Atkinson, Simona Petrescu, Adriana Baran, Daniela C. Culita, Ramona Ene, Bogdan Trica, Elena M. Anghel

**Affiliations:** 1 Institute of Physical Chemistry-Ilie Murgulescu of the Romanian Academy, 202 Splaiul Independentei, 060021 Bucharest, Romania; gpetcu@icf.ro (G.P.); irinaatkinson@yahoo.com (I.A.); simon_pet@yahoo.com (S.P.); adibaran@gmail.com (A.B.); dculita@icf.ro (D.C.C.); ene.ramona@yahoo.com (R.E.); 2 National Institute of Materials Physics, Atomistilor 405A, 077125 Magurele, Romania; nicoleta.apostol@infim.ro; 3 National Institute for Research & Development in Chemistry and Petrochemistry-ICECHIM, 202 Splaiul Independentei, 060021 Bucharest, Romania; trica.bogdan@gmail.com

**Keywords:** Ta/Me-MCM-41, Me (Nb, Ti, V, Co), bimetallic catalysts, photocatalysts, olefine oxidation, organic pollutants

## Abstract

Bimetallic (Ta/Ti, V, Co, Nb) mesoporous MCM-41 nanoparticles were obtained by direct synthesis and hydrothermal treatment. The obtained mesoporous materials were characterized by XRD, XRF, N_2_ adsorption/desorption, SEM, TEM, XPS, Raman, UV-Vis, and PL spectroscopy. A more significant effect was observed on the mesoporous structure, typically for MCM-41, and on optic properties if the second metal (Ti, Co) did not belong to the same Vb group with Ta as V and Nb. The XPS showed for the TaTi-MCM-41 sample that framework titanium is the major component. The new nanoparticles obtained were used as catalysts for oxidation with hydrogen peroxide of olefinic compounds (1,4 cyclohexadiene, cyclohexene, styrene) and photodegradation of organic pollutants (phenol, methyl orange) from water. The results showed improvementsin activity and selectivity in oxidation reactions by the addition of the second metal to the Ta-MCM-41 catalyst. The slow addition of H_2_O_2_ was also beneficial for the selectivity of epoxide products and the stability of the catalysts. The band gap energy values decreased in the presence of the second metal, and the band edge diagram evidenced positive potential for all the conduction bands of the bimetallic samples. The highestlevels of photocatalytic degradation were obtained for the samples with TaTi and TaV.

## 1. Introduction

Nanomaterials with ordered mesoporous structures have been widely used in the catalytic or photocatalytic oxidation of organic compounds from water. In this group of materials, MCM-41 materials modified by the inclusion of metals, either into the framework or well dispersed on the pores’ surfaces, are the most frequently studied due to their excellent properties, such as uniform pores hexagonally arranged, larges pecific surface area, and pore volume values [1,2,3,4]. Their narrow pore size distribution, high surface area, and pore volume make MCM-41s promising supports for metal and oxide catalysts. A key role in the textural, structural, and chemical properties of these materials is played by the synthesis method. The most extensively studied method is direct hydrothermal synthesis, i.e., directly adding a metal ion precursor to the synthesis gel prior to hydrothermal treatment. Therefore, many heteroatoms, such as Ti, V, Mo, W, Cr, Fe, Mn, Co, Ni, Ru, W, Nb, and Ta [5,6,7,8,9,10,11], have been incorporated into mesoporous silicas by this method, and the obtained materials have been largely used as catalysts [1,2,6,7,8,9,10,11,12], adsorbents [13], or photocatalysts for water decontamination [2,4,5,13,14,15]. The improved performance is attributed to the small loading of the isolated tetrahedrally coordinated metal oxide centers [12,16]. Thus, studies revealed that V, Nb, and Ta species supported silica, the predominant presence in isolated MeO_4_ species [17,18,19,20]. The selectivity in the oxidation reactions was attributed to the presence of Me-O-Si bonds in the catalyst structure. Raman results indicated that incorporating the Ta atom into the MCM-41 structure forms three types of tantalum oxide species: isolated TaO_4_ within MCM-41, isolated surface TaO_4_, and bulk Ta_2_O_5_. These species can coexist individually, and their relative peak intensity depends on the Ta concentration [18]. The catalytic properties of the surface TaO_4_ species are very different from those of the bulk Ta_2_O_5_ with acidic characteristics. Mesostructured oxides were obtained by associating tantalum oxide with various cations suchas Nb, Ti, Cu, Fe, or Ni [13,16,18,19,20,21,22,23,24,25,26,27]. Modified mesoporous oxides with tantalum [25,26,27,28,29], and especially Ta-MCM-41 [18,27,29], have proven to be active catalysts. However, tantalum is not among the most frequently used and studied metals supported on the surfaces of mesoporous materials, although these compounds have proven catalytic and photocatalytic activity. Active catalysts were thus obtained in the oxidation of styrene, phenol, and sulfides with H_2_O_2_ [25] or tetrahydroperoxide [27]. The supported tantalum catalysts, such as Ta-MCM-41, prepared by the grafting of Ta(OEt)_5_ on MCM-41 [28,29], and Ta_2_O_5_–SiO_2_, obtained by the sol–gel method [29] were active and selective in the epoxidation of styrene with tert-butyl hydroperoxide [28] and in the selective oxidation of a pyrimidine thioether [29]. Comparative studies on the catalytic properties of tetra- and pentavalent metal (Ti, V, Nb, Ta)-substituted molecular sieves indicated different properties of the immobilized metal species [30,31,32]. In addition to the properties of metal species, the immediate environment can strongly influence the oxidation reaction. It was demonstrated that in the cyclohexene epoxidation reaction, the active intermediates on Ti catalysts are the Ti-OOH species. In contrast, Nb and Ta catalysts react through Nb-(η^2^-O_2_) and Ta-(η^2^-O_2_) complexes [30]. These intermediates react with alkenes to yield epoxides. In contrast to Nb and Ta, V-substituted molecular sieves are poorly selective epoxidation catalysts, believed to operate via oxovanadium intermediates rather than through peroxymetal species [33]. Although all these studies highlighted the abilityof monometallic catalysts with tetra or pentavalent metals to activate H_2_O_2_ in the epoxidation of alkenes, an insignificant number of publications studied the properties of bimetallic catalysts obtained by immobilizing these metals on molecular sieves with ordered porous structures, such as MCM-41 mesoporous silica.

A further notable feature is the photocatalytic activity of tantalum-doped titanium dioxide in degrading dyes [26]. Dyes represent an enormous risk for the preservation of ecosystems, and for human health [24,26]. Even in low concentrations (e.g., 1.0 mg/L), they prevent light absorption in aqueous environments and decrease the photosynthetic activity and the available oxygen for local species. Additionally, dyes are toxic, mutagenic, carcinogenic, and non-biodegradable substances [33].

Although a limited number of works have studied the effect of Nb or Ti on the catalytic or photocatalytic activity of Ta immobilized on silica support [19,28], there are no comparative studies on the effect of transition metals such as V or Co on Ta immobilized on MCM-41, especially for bimetals immobilized by direct synthesis. Here, a series of single and binary metal oxide (Ta and Ti, V, Nb, or Co)-modified mesoporous MCM-41 photocatalysts were prepared using a well-known direct synthesis method with a hydrothermal treatment. The effect of the second metal on the structure, texture, optic properties, active species, and photocatalytic properties was evaluated. The newly obtained photocatalysts with mesoporous structures were used for catalytic oxidation with hydrogen peroxide (H_2_O_2_) of the olefinic compounds (1,4 cyclohexadiene, cyclohexene, styrene) and photodegradation of organic pollutants (phenol, dyes) from water.

## 2. Materials and Methods

### 2.1. Materials

The materials used for the synthesis of Ta/Nb, Ti, V, Co-MCM-41 mesoporous molecular sieves were as follows: tetraethyl orthosilicate (TEOS), cetyltrimethylammonium bromide (CTMAB), TaCl_5_, NbCl_5_, titanyl acetylacetonate, and Co(NO_3_)_2_·6H_2_O from Merck (Darmstadt, Germany), VOSO_4_·5H_2_O from Fluka (Buchs, Switzerland), sulfuric acid (98%), 1-propanol (CH_3_CH_2_CH_2_OH, ACS reagent, ≥99.5%), and ethanol (CH_3_CH_2_OH, ACS reagent, ≥99.5%) from Sigma-Aldrich (St. Louis, MO, USA), and sodium hydroxide (NaOH, ≥98%) from Lach-Ner s.r.o. (Neratovice, Czech Republic). Tantalum andniobium were stabilized with oxalic acid purchased from Merck (Darmstadt, Germany). The olefinic compounds purchased from Merck (Darmstadt, Germany), 1,4 cyclohexadiene (C_6_H_8_), 97%, cyclohexene (C_6_H_10_), ≥99.5%, styrene (C_8_H_8_), ≥99%, were oxidized with hydrogen peroxide (H_2_O_2_), 30%, Merck (Darmstadt, Germany), using acetonitrile (CH_3_CN), 99.8%, Merck (Darmstadt, Germany) as solvent. Methyl orange dye (85%) and phenol (≥99%) from Merck KGaA, Darmstadt, Germany were used for the photocatalytic reactions.

### 2.2. Photocatalyst Preparation

Ta, Nb, Ti, V, and Co species were incorporated into the mesoporous molecular sieves of MCM-41 type by direct synthesis and hydrothermal treatment. The metal precursor was added in situ to the synthesized silica gel. The molar ratio of Ta/Me was calculated to be 1. In the first step, 0.63 g CTMAB was dispersed in 35 g of deionized water (this mixture was named A). The pH of 1.5 was adjusted with sulfuric acid (98%), and the mixture was kept under continuous stirring for 1 h. Mixture B was obtained from TEOS solution (2.8 M) in ethanol-Et and 1-propanol-Pr (Et/Pr molar ratio = 6.5). After 10 min of stirring, into the alcoholic solution of TEOS were introduced TaCl_5_ and oxalic acid. After another 10 min, the aqueous solution of the metal precursor was added. When all the components had dissolved, mixture B was introduced dropwise into A under continuous stirring for another 1 h. Finally, the pH of the obtained sol was adjusted to 10.5 with an aqueous solution of NaOH (3 N). The aging of the sol–gel was carried out for 24 h at room temperature and for 5 days at 100 °C by hydrothermal treatment in Teflon-lined stainless steel autoclaves. The as-synthesized samples were filtered, washed with deionized water, dried at 100 °C for 6 h, and calcined for 6 h at 550 °C in airflow.

### 2.3. Materials Characterization

The obtained materials were characterized by XRD, XRF, N_2_ adsorption/desorption, SEM and TEM microscopy, Raman, XPS, UV-Vis, and PL spectroscopy.

X-ray diffraction (XRD) analysis was performed using a Rigaku Ultima IV diffractometer (Rigaku Corp., Tokyo, Japan) with Cu Kα, λ = 0.15406 nm. Phase evaluation was made with the help of Rigaku PDXL (Version no 1.8, Rigaku, Tokyo, Japan) with the Whole Powder Pattern Fitting (WPPF) module, connected to the database ICDD-PDF-2.

Using a Rigaku ZSXPrimus II spectrometer (Tokyo, Japan), XRF elemental analysis of the samples was carried out under vacuum. Combining the EZ-scan with the Rigaku SQX fundamental parameters software (Version 5.18, Rigaku, Tokyo, Japan) (standardless), which can automatically correct all matrix effects, including line overlaps, allows for an analysis of the test results.

Micromeritics ASAP 2020 instrument (Norcross, GA, USA) was utilized to perform N_2_ physisorption analysis on the samples for textural characterization. Before every measurement, the samples were degassed for five hours at 300 °C under vacuum. The BET model was used to calculate the apparent surface areas from the adsorption branches, while the amount of nitrogen adsorbed at the relative pressure of 0.99 was used to calculate the total pore volume. The micropore and mesopore volumes and particle surface areas were derived by the t-plot method.

The morphology and microstructure of the samples were explored by scanning electron microscopy (SEM) FEI Quanta 3D FEG (FEI, Brno, Czech Republic) and transmission electron microscopy (TECNAI 10 G2-F30 and F20 G2 TWIN Cryo-TEM-FEI, Eindhoven, The Netherlands).

The UV-Raman spectra were collected using anHR800 spectrometer (HORIBA FRANCE SAS, Palaiseau, France) equipped with a He-Cd laser (325 nm), a CCD detector, and 2400 gr/mm gratings. The laser beam excited a sample spot of ~0.8 μm through a 40×/0.47 NUV microscope objective from Olympus (Olympus Corporation, Tokyo, Japan). The laser power was kept below 0.5 mW at the sample position to avoid laser-induced changes. The entrance slit width was adjusted to 200 µm. The background corrected spectra were fitted with a Lorenz–Gaussian profile by PeakFit 4.12 software.

DR-UV–vis spectroscopy was used to detect coordination states of 3D metallic species (Co, Nb, V, Ti) added to Ta-MCM-41 by direct synthesis. The spectra of the samples were recorded in the range of 200–850 nm using a JASCO V570 spectrophotometer (Tokyo, Japan). The band gap energies of all the samples were obtained for indirect transitions from Tauc’s plot using the Kubelka–Munk function [34]. Since it is difficult to split into components a Kubelka–Munk spectrum for composite photocatalysts, a simplified procedure (baseline method) reported in the literature [34] was used.

For the XPS measurements, the AXIS Ultra DLD installation (Kratos Surface Analysis, Manchester, UK) was used, using Al Kα1 radiation (1486.74 eV) produced by a monochromatized X-ray source with a power of 144 W (12 kV × 12 mA). The high-resolution spectra were recorded using the “hybrid lens” mode, with a pass energy of 40 eV and a “slot” aperture. The binding energy scale was calibrated to the “standard” C 1s value of 284.6 eV, and the spectra of the levels of interest were analyzed using Voigt profiles, methods described in ref. [35].

The photoluminescence spectra of the samples were obtained using an FLSP 920 spectrofluorimeter (Edinburgh Instruments, Livingston, UK) with an Xe lamp as an excitation source (λ_exc_ = 320 nm). For all measurements, excitation and emission slits were 7 nm.

Oxidation of the olefinic compounds with hydrogen peroxide was carried out simultaneously in 5 thermostated microreactors (65 °C), each using 0.05 g of catalyst. Acetonitrile was used as asolvent. The molar ratio of organic compound/solvent/hydrogen peroxide was 1/1.8/3. Hydrogen peroxide was added on the first time of thereaction or, dropwise, during the first 3 h of the reaction (slow addition). The hot filtration experiments were performed by separating the catalyst from the reaction mixture after 5 h of reaction time, and the filtrate was then kept at the reaction temperature for anadditional 48 h. The reaction products were filtered through Millipore membrane filters and analyzed on a DANI GC 1000 gas chromatograph with a metal capillary column using a flame ionization detector (FID). Leaching during the reaction was verified. Thus, the catalyst was recovered from thereaction medium, washed with acetonitrile, separated by centrifugation, dried at 80 °C, reactivated in theair at 350 °C to remove the possible adsorbed compounds [9], and reused in thereaction.

The photocatalytic activity of modified Ta-MCM-41 samples was evaluated in oxidative degradation of phenol (Ph) in aqueous solution (0.002 M) and methyl orange dye (MO)concentration of solution 1 × 10^−5^ M. The photocatalytic tests were conducted under stirring in a closed room at 30 °C by adding 2 mg of the photocatalyst in 10 mL aqueous reactant solution. The reaction mixture was stirred in darkness for 30 min to allow the adsorption of organic compounds from thesolution on the surface of thephotocatalyst. Further, a UV mercury lamp (60 W) with afilter of 254 nm was used for irradiation. At certain spans, 2 mL of the mixture was taken out, and the photocatalyst was separated by centrifugation and, further, using a Millipore syringe filter of 0.45 μm. The filtered dye solution was spectrophotometrically measured using the same JASCO V570 UV–vis spectrophotometer. The photocatalytic degradation efficiency was expressed as C_t_/C_0_, where C_t_ is the solution concentration at 1, 3, or 5 h and C_0_ is the initial concentration of MO or Ph at t = 0.

## 3. Results and Discussion

### 3.1. Characterization of Materials

In this study, bimetallic MCM-41 ordered mesoporous molecular sieves with Ta/Nb, Ta/V, Ta/Ti, and Ta/Co were successfully prepared. The XRF-derived percentage for each metal in the obtained samples is listed in Table 1. A significant decrease in theTa/Me molar ratio for the TaTi and TaCo samples is noted. In contrast, for the samples with Nb and V, obtained using similar synthesis conditions, this ratio is closer to 1, the value calculated for these syntheses.

**Figure 1:**
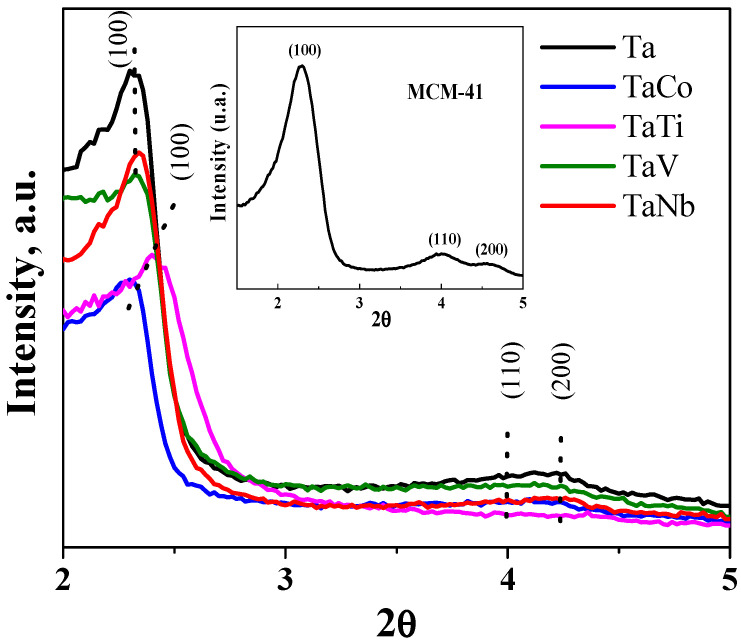
The low-angle XRD patterns of the obtained nanomaterials compared to that of the MCM-41 mesoporous silica (inset).

The significant decrease in the Ta/Me molar ratio observed for the samples in which the second metal is not from the Vb group (Ti, Co) may be due to the greater capacity of Ti and Co for incorporation into the silica network highlighted in multiple other publications [6,16,36,37]. Figure 1 shows the XRD diffractograms of the Ta-MCM-41 and Ta/Me-MCM-41 samples recorded at low angles. The diffractograms were compared to MCM-41 mesoporous silica (inset in Figure 1). The diffractograms reveal a distinct and sharp diffraction peak corresponding to the plane (100), along with weaker and broadening secondary diffraction peaks represented by the (110) and (200) planes. However, when a second metal near Ta is added to the MCM-41 structure, the intensity of the first diffraction peak decreases while the secondary peak declines or vanishes, indicating structural distortions caused by metal incorporation [36]. This effect is influenced by both the synthesis method [9] and by the properties of the immobilized metal. This effect is more significant in the case of metals from theVb group [8].

Dubiel et al. [37] observed a similar intensity trend for Ti and Fe incorporated into MCM 41. Table S1 summarizes the position of the diffraction peak for the (100) plane, the interplanar spacings (d100), and the lattice parameters (a_0_). The peak position for the (100) plane is slightly shifted to a lower value for the TaCo sample, which has a significantly reduced peak intensity corresponding to the (100) plane and lower surface area. However, a slight shift for higher 2θ values compared to the Ta sample for TaTi, TaV, and TaNb was observed, suggesting the incorporation of the second metal into the Ta-MCM41 framework. Jankowska et al. [38] also reported a shift of the (100) diffraction peak toward higher 2θ values for bimetallic MCM-41systems (Cu-Fe, Cu-Mn, and Fe-Mn), possibly due to the deposition of transition metal species within the pores, leading to a reduction in their size. A similar shift was observed for Nb, Ta-MCM-41 obtained by hydrothermal direct synthesis in [19]. The cell parameter a_0_, corresponding to the point equidistant between adjacent parallel pores [39], was calculated using the 2θ position of the maximum intensity peak (d100) and interplanar spacing (d) with the formula a_0_= 2d100/√3. The values calculated for the cell parameters a_0_ ranging between 3.67 nm and 3.83 nm are slightly lower than the value obtained for the Ta-MCM 41 sample, depending on the type of the incorporated metal and its content.

**Figure 2:**
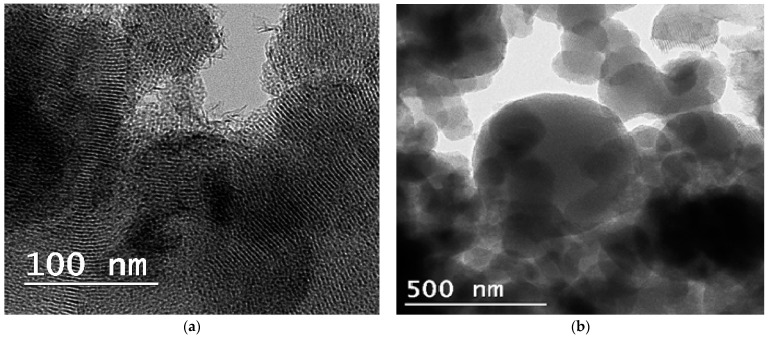
TEM images of the hexagonal channels (**a**) and nanoparticles (**b**) of Ta-MCM-41 sample typical for MCM-41 materials.

Additionally, the high-angle XRD patterns (Figure S1) showed no characteristic peak regarding crystalline metal (Nb, V, Ti, Co) species oxides, only a broad peak at around 2θ = 23°, which proves the presence of amorphous species of silica support. The TEM images of the Ta-MCM-41 (Figure 2a) and Ta/Me-MCM-41 (Figure S2) samples indicate the presence of materials with ordered porous structures in a high percentage with cylindrical pore channels, confirmed in a hexagonal array. In Figure 2b, the TEM image shows mostly spherical nanoparticles with dimensions close to 100 nm. Many of these nanoparticles are agglomerated in spherical packages or wire-like shapes.

**Figure 3:**
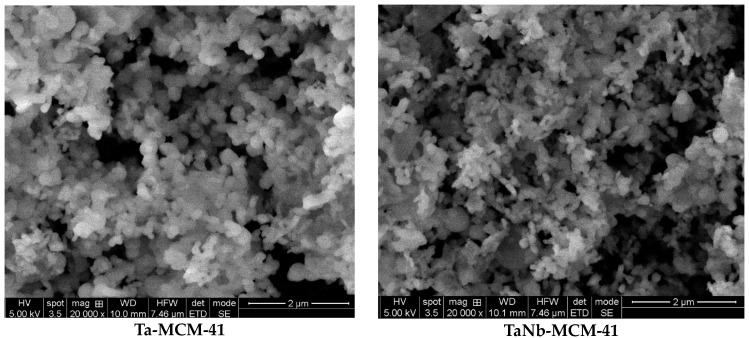

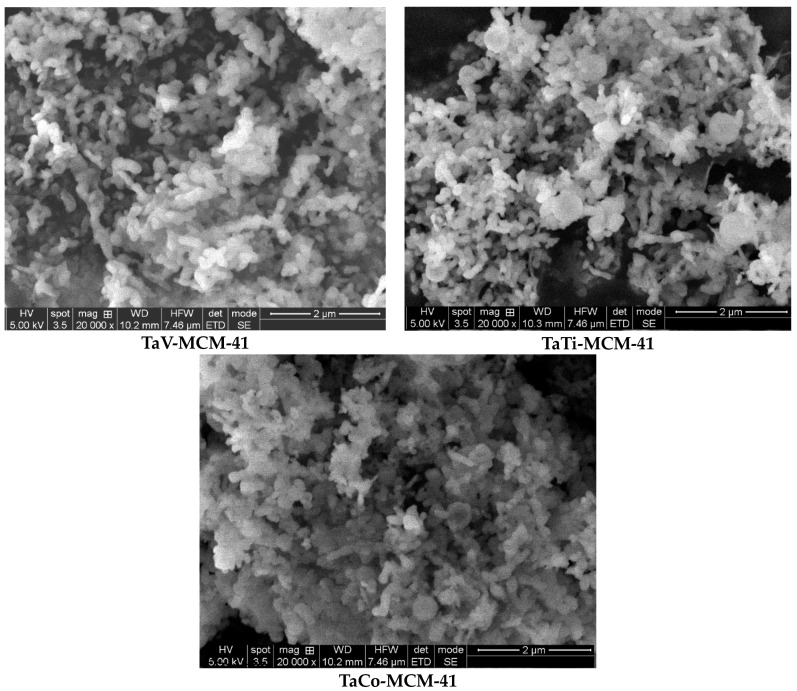
SEM images of the Ta and bimetallic Ta (Nb, V, Ti, Co) samples.

The SEM microscopy images of the obtained samples (Figure 3) confirm the spherical morphology of the agglomerated nanoparticles. In the bimetallic samples, wire-like shapes may also be present.

**Figure 4:**
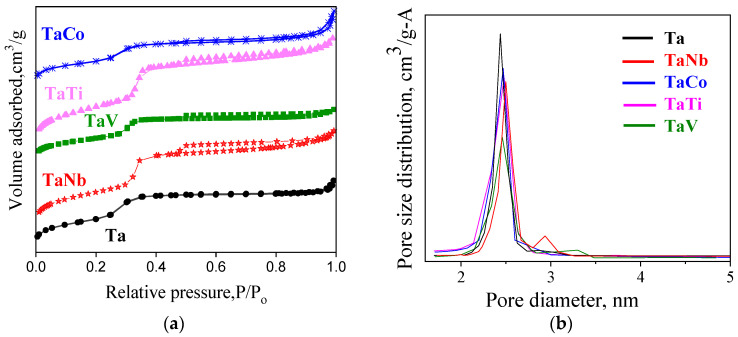
N_2_ adsorption–desorption isotherms (**a**) and pore size distributions (**b**) of Ta/Me-MCM-41 modified samples.

The type IV nitrogen physisorption isotherms characteristic of MCM-41 mesoporous silica [8,9] are illustrated in Figure 4a for the bimetallic and Ta-MCM-41 catalysts. The hysteresis loop of isotherms is insignificant, except forin the TaNb sample. H4-type hysteresis was detected for the latter sample, indicating that larger pores are also present (Figure 4b). The insignificant changes in the pore diameter and the absence of hysteresis, typical for MCM-41 materials [9], can be due to well-dispersed metallic species in the silica mesoporous network and on the pore surface. Figure 4b shows a narrow pore size distribution for all the samples. The volume of the pores with larger sizes is insignificant even in the case of the TaNb sample. Table 2 presents the variation of the specific surface area, pore volume, and pore sizes of the obtained samples. These materials showed a large surface area and pore size diameter similar to those reported for Me-modified MCM-41 mesoporous materials [8,9].

The insignificant changes in the pore diameter may be due to well-dispersed metallic species in the silica mesoporous network and on the pore surface.

**Figure 5:**
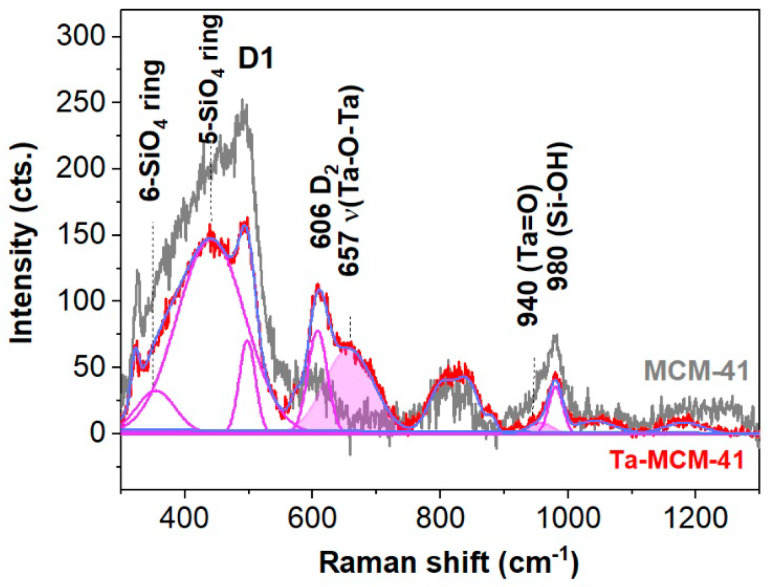
Fitted UV–Raman spectra of Ta-MCM-41 samples (blue line stands for global fit, R^2^ = 0.9902. Some components were left out for clarity). The MCM-41 spectrum is represented for comparison, especially for the wide band at about 800 cm^−1^ (Si-O-Si stretching modes), similar to the two spectra illustrated.

UV–Raman spectroscopy is a valuable tool instudying extra- and framework transition metal ions, as well as microporous and mesoporous framework and highly dispersed transition metal oxides on supports [40,41]. This arises from the charge transfer between the framework oxygen anion and the framework transition metal ion. Moreover, fluorescence caused by the impurities (coke, organic impurities, surface defects, etc.) in mesoporous materials can be avoided by using UV exciting lasers. The Raman spectra up to 650 cm^−1^ of the monometallic (Ta, Nb, V, Ti, Co)MCM-41 samples (Figure S3 and Table S2 [42,43,44,45,46,47,48,49,50]) are dominated by the spectra features of the n-membered SiO_4_ rings (where n is within 3–7) of the MCM-41 [42,43]. The 490 and 606 cm^−1^ bands belong to the defect bands of the four- and three-membered SiO_4_ rings. The Raman modes for the four-membered SiO_4_ rings are shifted towards lower wavenumbers at 481 cm^−1^ under the vanadium influence [45]. The distinct band at about 657 cm^−1^ in the Ta-MCM-41 (Ta) spectrum belongs (Figure 5) to the Ta-O-Ta (TaO_6_) stretching mode in crystalline Ta_2_O_5_ [46], which is the major tantalum phase. Furthermore, octahedral coordinated cobalt gave a band at about 664 cm^−1^ in the Co-MCM-41 spectrum. The 808 cm^−1^ band is due to the symmetric stretching modes of the SiO_4_ tetrahedra in the MCM-41 [42].

When metal oxides are incorporated in theMCM-41 framework, spectral modifications are expected within the 900–1200 cm^−1^ range due to stretching Me/Si-O bonds in a tetrahedral coordination TO_4_ [42,43]. Thus, the weak band of the polymerized TaO_x_ with TaO_4_ coordination [46] on the surface of the MCM-41 was noticeable by fitting the Ta spectrum at about 940 cm^−1^ (see Figure 5). Previous ^29^Si MAS NMR studies [29] highlighted the incorporation of tantalum into the sol–gel-obtained silica framework by (–O–Si)_3_–Ta=O bonds due to modification of the Q^4^/Q^3^ ratio in comparison with the silica counterpart (Q^4^ and Q^3^ represent the SiO_4_ tetrahedra with 0 and 1 non-bridging oxygen atoms, NBOs). The V-MCM-41 spectra show a strong band at about 980 cm^−1^ (Figure S3a), possibly due to the stretching of the SiO-H bonds [50]. Since the SiO-H band within the range of 3740–3750 cm^−1^(isolated hydroxyl groups on the MCM-41 support [50]) is less intense than those of the Me-MCM-41 spectra in Figure S3b, the assignment of the 980 cm^−1^ might consist of stretching vibrations of Si-O-Si in SiO_4_ tetrahedra [51] with two or on NBOs, e.g., Q^2^ or Q^3^ units [52]. The intense band at about 1100 cm^−1^ in the Ti-MCM-41 spectrum indicates complete incorporation of the tetrahedrally coordinated titanium ion in a flexible environment [48] by Si replacement at its sites of MCM-41. The more flexible the environments, the greater the shifting to the lower wavenumbers compared to the 1125 cm^−1^ band position assignable to Si-O-Ti for the TS-1 [48]. Raman spectroscopy is useful for hydrogen bonding interactions between catalysts and water [50]. Additionally, the presence of the isolated hydroxyl groups on the MCM-41 support [50] is validated by the small, sharp band at about 3740 cm^−1^ in Figure S3b. Furthermore, the MCM-41 framework retained free water, as depicted inits broad band peaking up at 3490 cm^−1^(Figure S3b). The hydroxyl groups linked to the metal ions give weaker Raman modes at about 3600 cm^−1^, namely the left-tailed shoulders of the SiO-H band [50]. The small band at about 3610 cm^−1^ (Figure S3b) signals the presence of the Me-OH [49,50] and free water [50].

**Figure 6:**
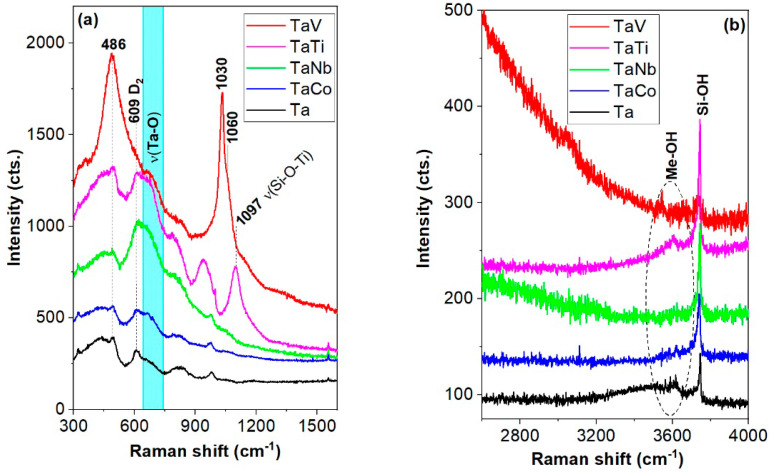
(**a**,**b**) UV–Raman spectra of the Ta and Ta(Co, Nb, Ti, V) samples.

**Figure 7:**
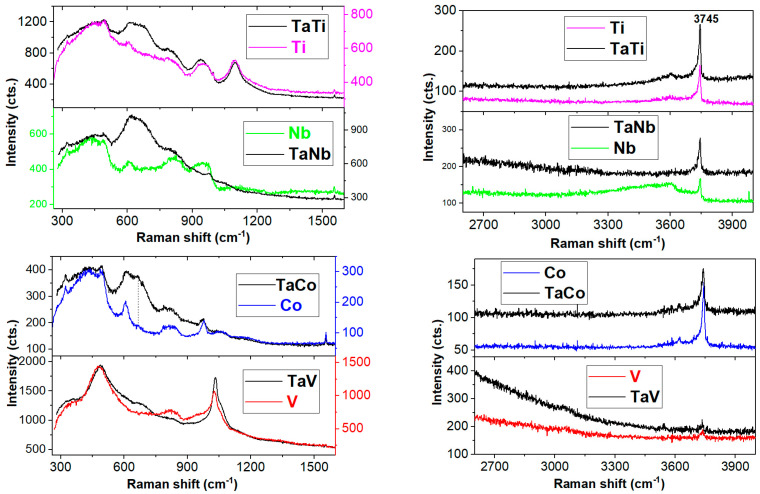
Comparative UV–Raman spectra of monometallic (Ta, V, Ti, Nb, Co)MCM-41 and bimetallic Ta(V, Ti, Nb, Co) samples.

The bimetallic Ta(V, Co, Nb, Ti) spectra show distinct spectral features within the 640–745 cm^−1^ range, unlike their (V, Co, Nb, Ti)MCM41 counterparts (Figure 6 and Figure 7, Tables S2 and S3). This behavior is due to the vibrations of the TaO_6_ bonds (see Table S3) [18,26,42,43,44,45,46,47,48,49,50,51,52,53,54,55,56]. The fitting of the peak within the range of 554–758 cm^−1^ for the bimetallic TaTi spectrum revealed three components, namely 607, 616, and 685 cm^−1^. The 616 cm^−1^ band might be attributable to the A_1g_ modes of the extra-framework TiO_2_ as rutile [43]. A true resonant effect was expected for the TaCo sample due to its UV absorption at about 320 nm, which is very close to the Raman excitation line of 325 nm. The latter spectrum in Figure 7 exhibits notably intense, split bands peaking around 670 and 707 cm⁻^1^. These peaks are likely to be attributable to tantalum and cobalt oxides, probably in octahedral coordination. The least intense peak within the 640–745 cm^−1^ region (vibration modes of the tantalum speciation) seems to be recorded for the TaV spectrum in Figure 7. This is a consequence of the very intense band at the 486 and 1030 cm^−1^ bands (~5 times stronger than the other bimetallic spectra) due to a nearly resonant effect (the UV–vis absorptions at about 262 and 380 nm of the MCM-41 framework and supported vanadium, respectively, are presented further in the UV–vis spectra). Samek et al. [57] reported that UV–Raman spectroscopy is more sensitive to the isolated and less polymerized VO_x_ species, unlike vis–Raman spectroscopy.

The 1030 cm^−1^ and 486 cm^−1^ bands of the TaV spectrum (V=O stretching modes and 4-membered TO_4_ units, where T stands for the tetrahedrally coordinated Si or V) indicate incorporation in the MCM-41 framework. The shoulder at about 1060 cm^−1^ might originate from the shorter V=O bonds [56]. Moreover, the presence of multiple narrow spectral features above 1000 cm^−1^ is due to various surface VO_x_ species and increasing vanadia surface coverage [57]. According to the intense 486 cm^−1^ band, very abundant four-membered rings of SiO_4_were present in the TaV sample. Clear evidence of the Ti’s incorporation as TiO_4_ units is the strong band at about 1097 cm^−1^ in Figure 7. Conversely, the amount of tetrahedral units with Nb/Si-O bonds within the range of 800–1200 cm^−1^ is rather poor in the TaNb compared to the Nb spectrum. The lack of a935 cm^−1^ band in the TaNb spectrum in comparison with the Nb spectrum indicates that surface-polymerized niobia species [46] were absent. Given the intense band at 3744 cm^−1^, the 978 cm^−1^ band is very likely to be assignable to the Si-OH, although the presence of isolated NbO_4_ is not ruled out.

The tinny Si-OH band at 3738 cm^−1^ (Table S2) for the TaV spectrum indicates that the TaV sample is nearly as desiccated as the V-MCM-41 sample. This is also supported by the lack of a ~970 cm^−1^ band for Si-OH stretching vibrations in the TaV spectrum. The bimetallic TaNb spectrum has a wide band peakingup at about 945 cm^−1^ originating from the (Ta, Nb)-O-Si stretching [46]. A less hygroscopic TaNb sample than the Nb-MCM-41 sample is presented in Figure 7. The pentavalent elements (Ta, Nb, and V) were reported to form (O=Me^5+^ OSi)_3_OH)Si(OH)_2_ and (O=Me^5+^ (OSi)_3_)Si(OH) [31] in hydrated samples. However, almost desiccated TaV samples were obtained. Additionally, Nb^5+^ tetrahedral moieties in the MCM-41 framework were reported for the bimetallic AlNb-MCM-41 materials [58] by NMR and UV–vis spectroscopies. The amount of tetrahedral Nb is reduced in the current work.

**Figure 8:**
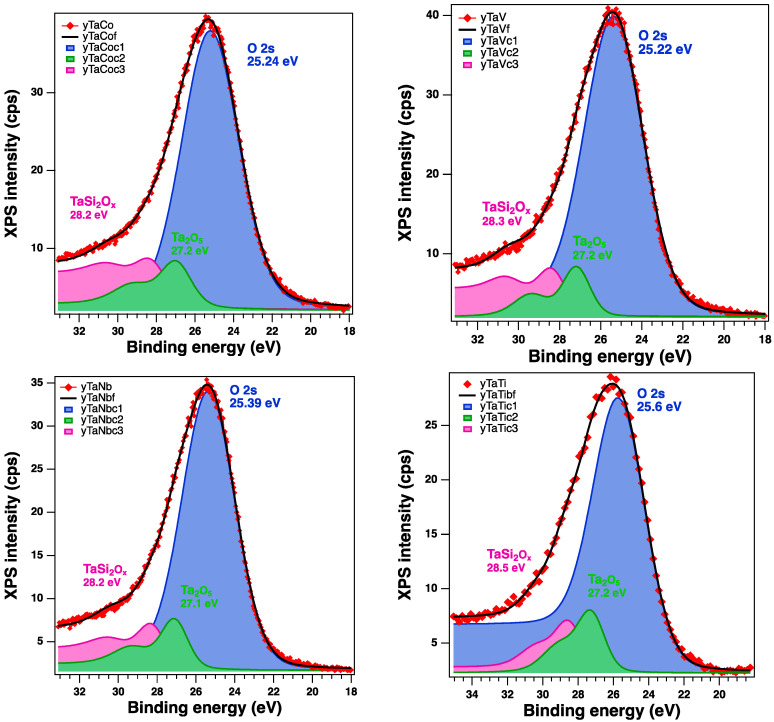
Fitted XPS high-resolution Ta4f and O2s spectra of the bimetallic Ta/Me-MCM-41 samples (red symbols—raw data, black line—fit, blue, green and lavender solid colors—the main compounds identified).

**Figure 9:**
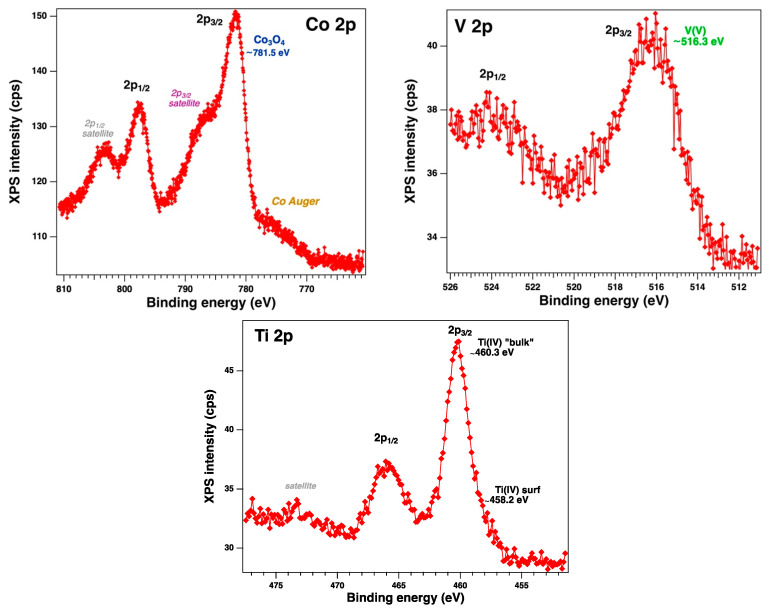
XPS spectra of Co2p, V2p, and Ti2p for the TaCo-MCM-41, TaV-MCM-41, and TaTi-MCM-41 samples.

XPS was further utilized to analyze the chemical states of elements from the bimetallic materials’ (TaCo-MCM-41, TaV-MCM-41, TaTi-MCM-41, and TaNb-MCM-41) surfaces. The wide survey spectra of the Ta/Me-MCM-41 samples showed (Figure S4) that all the main elements (O1s, Si2p, Co2p, V2p, Ti2p, Nb3d, Ta4f) could be detected. Unfortunately, the peak intensity of the Ta4f and Nb3d XPS spectra for the bimetallic samples is very low (Figures S5 and S6). In addition, the peak of the Ta4f is masked by that of O2s under abundant oxygen conditions, and at the same time, a lower amount of Ta is highly dispersed into the silica support. In Figure 8 are presented the high-resolution Ta4f and O2s spectra of the bimetallic Ta/Me-MCM-41 sample. The features of the deconvoluted spectra show BE values around 25 eV, 27 eV, and 28 eV that can be attributed to O2s, Ta_2_O_5_, and TaSi_2_O_x_ species, respectively. The previous studies evidenced changes in coordination for the supported metal oxides of the Group V metals. Thus, the surface tantalum species possess TaO_4_ coordination for low surface coverage and highly distorted TaO_5_/TaO_6_ coordination at intermediate and high surface coverage [59]. Therefore, the isolated surface TaO_4_ species are present at low surface coverage, especially on the SiO_2_ support. Additionally, on the silica surface, the maximum achievable density of Ta is much lower than on other oxide supports (~1 to 5–6 Ta atoms/nm^2^). Thus, the molecular structures and densities of the tantalum and niobium species on the surface are very similar. Research data [31,60] reported a lower density than 2 Nb atoms/nm^2^ for niobia-supported silica. These data could explain the low intensities observed for Ta4f and Nb3d in the XPS spectra from Figures S5 and S6. The use of Density Functional Theory (DFT) to investigate Ta oxides in various oxidation states evidenced that stoichiometry and amorphous states change the Coulomb repulsion and, hence, the binding energies [61]. The results demonstrated a substantial presence of undercoordinated Ta in all the samples. The longer Ta-O distances in the amorphous TaOx resulted in lower Coulomb repulsion and higher binding energies. These findings help to explain the difficulties in analyzing XPS spectra for both Ta and other oxide species, which were incorporated into MCM-41 mesoporous silica in smaller quantities through direct synthesis [62,63]. Previous research [12] has also shown that the mesoporous silica network of MCM-41 facilitated electron transfer between supported metals and the generation of oxygen vacancies by the incorporation of Vb metals. The Nb3d spectra from theTaNb-MCM-41 sample (Figure S6) can be attributed to the high dispersal of Nb_2_O_5_ into theMCM-41 network. The Co2p, V2p, and Ti2p XPS spectra of the bimetallic Ta (Co, V, Ti) samples are illustrated in Figure 9.

The analyzed spectra of Co 2p, V2p, and Ti2p revealed the coexistence of different chemical states. The Co2p spectrum presents two main components and their satellites, indexed as Co 2p3/2 and Co 2p1/2, attributed to Co_3_O_4_, which is a mix between 2+ and 3+ oxidation states of cobalt [64]. The spectrum of V2p shows a main peak at around 516 eV and the presence of the satellite at ~523 eV, attributed to V_2_O_5_. These results agree with other published results [65] that evidenced two main different coordinated forms for the isolated vanadium species on supports: tetrahedral V^5+^ of type VO_4_^3−^ and tetrahedrally coordinated vanadium sites ((SiO)_3_V=O). The XPS spectrum of the Ti 2p region shows titanium as tetrahedrally coordinated Ti^4+^ in the TaTi-MCM-41 sample. Therefore, the XPS Ti2p spectrum denotes (Figure 9) the most prominent peaks at 460.3 eV and 465.7 eV, which correspond to Ti 2p3/2 and Ti 2p1/2, respectively, from the framework [66]. Furthermore, the shoulder from 458.2 eV confirms that the extra-framework Ti is found in low concentrations.

The XPS O1s high-resolution spectra evidence (Figure S7) a higher intensity for the samples that contain only metals of the Vb group (TaV, TaNb) and a small shift towards lower BE values in the case of the sample with Ta and Ti. The binding energy at around 532 eV was attributed to lattice oxygen from the MCM-41 network and the shift towards ~533 eV to oxygen vacancies or less adsorbed oxygen species from the surface. In addition, in the case of the TaTi-MCM-41 sample, the XPS Si2p spectrum (Figure S8) indicates slightly lower intensity, with a small shift towards low BE values. We can thus consider that the high Si substitution with Ti in the silica framework influences the binding energy of both oxygen and silica.

**Figure 10:**
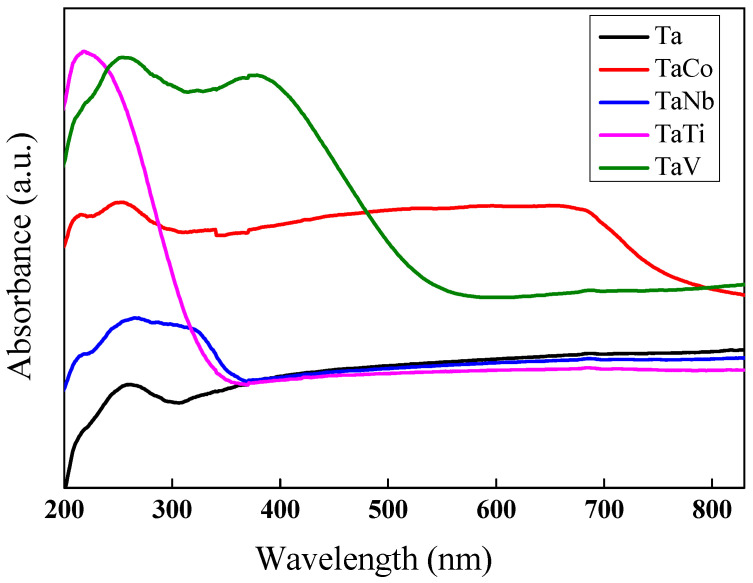
Diffuse reflectance UV–vis spectra of Ta-MCM-41 sample and Ta-MCM-41 modified with Co, Nb, Ti, and V by direct synthesis.

The UV–vis diffuse reflectance spectra of the synthesized samples are shown in Figure 10. All the spectra have an absorption band at around 263 nm, associated with the charge transfer from oxygen ions of theMCM-41 framework to the metal in tetrahedral coordination [13].

Two other intense absorption bands were observed for TaNb and TaV, at around 318 nm and 380 nm, respectively. Their appearance indicates the presence of penta- or hexacoordinated niobium (V) [19], and vanadium (V) [67] species in the modified materials. The TaV sample exhibited a broad band between 250 and 550 nm, due to the charge transfer associated with V-O electron transfer for tetrahedrally coordinated V^5+^ species. It corresponds to the ligand-to-metal charge transfer (LMCT) from oxygen to tetracoordinated titanium in isolated tetrapodal (Ti(OSi)_4_) or tripodal (such as Ti(OH)(OSi)_3_) units. The presence of this absorption band suggests the successful incorporation of Ti as anisolated species into the silica structure. Furthermore, the shoulder located at a higher wavelength (~270 nm) indicates the presence of more highly coordinated Ti species (penta- or hexacoordinated), which could occur through hydration by binding water molecules as extra ligands [68].

Ta-MCM-41 modification with the transition metals Co, Nb, V, and Ti by direct synthesis led to a red shift of the absorption spectra in all cases. Thus, the synthesized materials became active under visible light irradiation by lowering the energy of the band gap considerably compared to the sample Ta-MCM-41, as shown in Table 3 and Figure S9.

**Figure 11:**
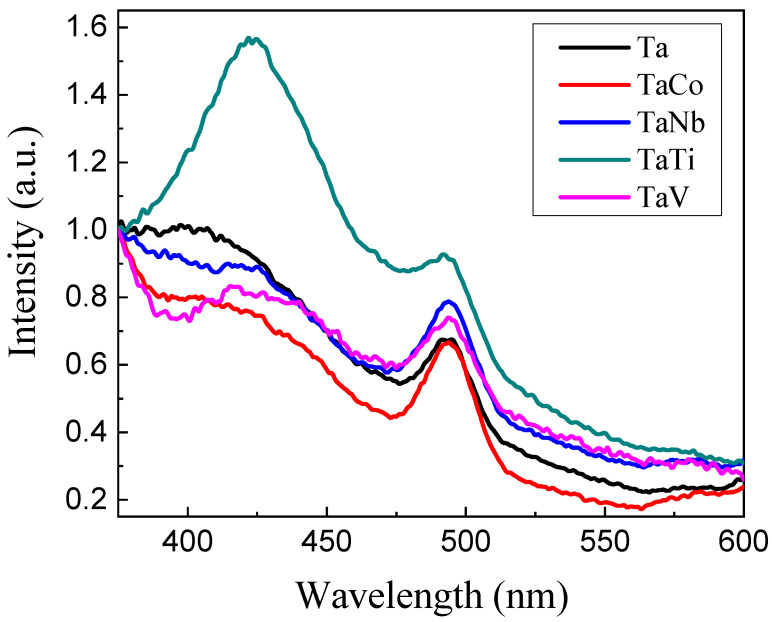
PL spectra of the obtained samples.

The photoluminescence (PL) spectra (Figure 11) were determined to evaluate the separation and recombination of the photogenerated charges in Ta-MCM-41 and Ta/Me-MCM-41 samples. The PL peak intensity is influenced by surface and internal defects. All the spectra exhibit two emission peaks at around 425 and 490 nm. The highest intensity of both peaks was obtained for the TaTi-MCM-41 spectra. This is the effect of Ti’s incorporation into the MCM-41-type silica network, which can induce structural disorders. These were confirmed by the X-ray diffractogram at the small angle of this sample (Figure 1). Additionally, the existence of the framework Ti was confirmed by the Raman and XPS results (Figure 7 and Figure 9). An increase in PL spectrum intensity due to structural disorders has also been previously reported [69]. The first peak was attributed to the band-to-band direct transitions. The intensity of this peak decreases in the order of TaTi > Ta > TaNb > TaV > TaCo due to higher electron–hole separation, which gives long-lived photogenerated charge carriers [70,71]. Furthermore, a shift from 400 nm to 425 nm can be observed for the bimetallic samples.

At the same time, a shift to 400 nm can be observed for the bimetallic samples. The second excitonic PL peak (490 nm) suggests that the samples contain high levels of defects generated by metal incorporation. Surface defects refer primarily to the metal ions dispersed on the silica surface and the incomplete coordination of surface species that induce oxygen vacancies. Thus, recombination centers for charge carriers are created. Furthrmore, the high dispersion of metal ions and the possibility of incorporating them into the silica network generate defects that act as donors or acceptors [22,32]. The peak with the highest intensity was obtained for the TaTi-MCM-41 sample. It is also confirmed for this sample that more defects influence its optical properties.

### 3.2. Catalytic Properties

**Figure 12:**
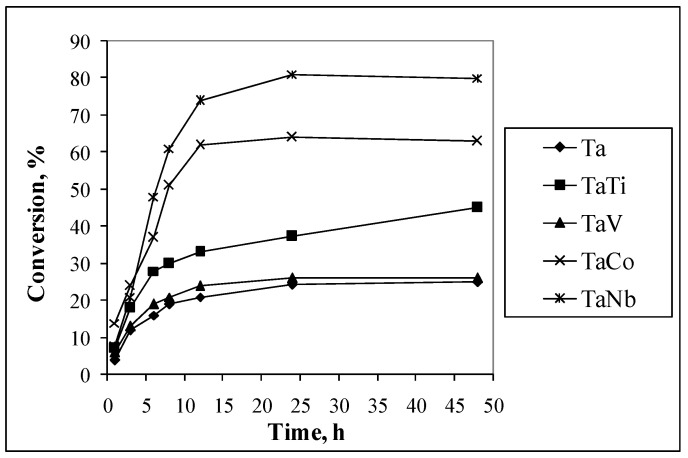
Conversion of 1,4 cyclohexadiene as a function of time reaction (slow addition of H_2_O_2_).

All the obtained materials are active and selective in the oxidation with H_2_O_2_ of the olefinic double bands. The oxidation of organic molecules under mild conditions is a topic of great interest [24,26,27,28,72]. A higher conversion was obtained in theoxidation of 1,4 cyclohexadiene on TaCo and TaNb catalysts (Figure 12). The increase in conversion was also evidenced for the TaTi sample.

**Figure 13:**
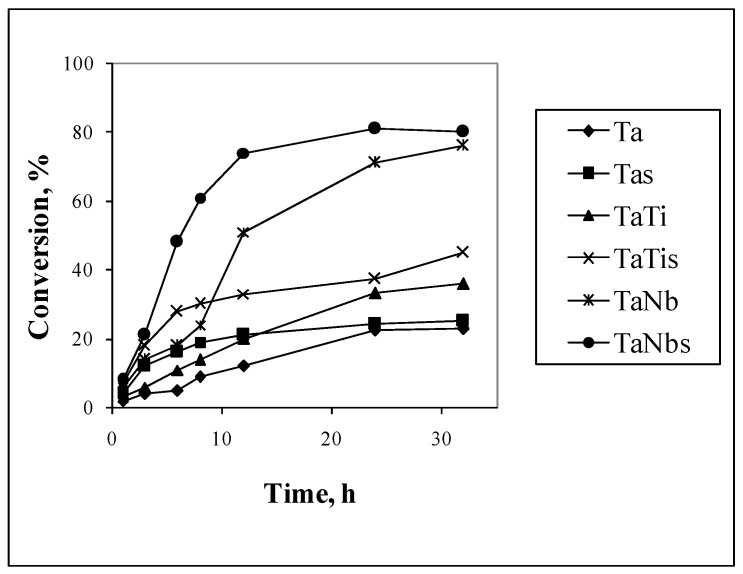
Effect of H_2_O_2_ addition on conversion of 1,4 cyclohexadiene.

The activity and epoxide selectivity of the Ta mesoporous molecular sieves (Table 4) were influenced by the catalyst composition and method of H_2_O_2_ addition. The effect of the slow addition of H_2_O_2_ is evidenced in Figure 13. It can be seen that the conversion for the samples marked with s (slow addition of H_2_O_2_ during the first 3 h of the reaction) is higher compared with those reactions in which the entire amount of hydrogen peroxide was added at the beginning of the reaction. These results indicate that the adsorption is a limitative step in the oxidation reaction.

The main reaction products were as follows: cyclohexenone and cyclohexenol for oxidation of 1,4-cyclohexadiene; cyclohexene oxide, cyclohexenone, and cyclohexenol for oxidation of cyclohexene; and benzaldehyde and styrene oxide in case of styrene oxidation. The second metal and slow addition of H_2_O_2_ favor the selectivity for epoxide. The selectivity for epoxide (cyclohexenol (HOL), cyclohexene oxide (HO), and styrene oxide (SO)) was high in the oxidation of cyclohexene and styrene for all the bimetallic catalysts. Higher selectivity for cyclohexanol was obtained for all the catalysts (Table 4) in 1,4-cyclohexadiene (Table 4). The addition of the second metal significantly increased both conversion and the selectivity for the epoxide (cyclohexene oxide (HO) and styrene oxide (SO)).

Figure 12 and Figure 13 and Table 4 show that the Ta-Nb and Ta-Co couples were more active in the olefin oxidation. These properties are known for both Ta and Nb compounds [25,27,28,29]. The activity of cobalt, titanium, and vanadium oxides in oxidation reactions is also known [6,8,9,11,12,14,28]. However, until now, the number of studies on bimetallic Ta-Ti, Ta-V, and Ta-Co catalyst activity in oxidation reactions has been insignificant. The obtained results presented in Table 4 were compared with data from the literature for catalysts obtained by immobilizing similar metals on silica-based supports (Table 5).

Higher values and selectivity towards epoxide are observed for most of the bimetallic catalysts studied. The highest conversion values were obtained in the oxidation of styrene and the highest selectivity for epoxide in the oxidation of cyclohexene. Theactivity of the prepared bimetallic catalysts was attributed to the metal species (MO_4_ or O=Me^5+^ OSi where M=Ti, V), highly dispersed metal oxide and the environment created by the silica with an ordered mesoporous structure. The indicated oxide species were identified using Raman, XPS, and UV–vis spectroscopies.

### 3.3. Photocatalytic Properties

Ta-based photocatalysts have also garnered considerable attention in photocatalytic applications due to their electronic structure and high chemical stability [13,16,22,78,79]. Thus, the photocatalytic properties of tantalum-based mesoporous materials were tested in the oxidative degradation reaction of phenol (Ph) and methyl orange (MO). These organic compounds, particularly phenol, and dyes, are significant as they are the main representatives of common pollutants found in wastewater [80,81,82]. For a better understanding of the effect of Ta-MCM-41 modification with different 3d metals (Co, Nb, V, Ti) on photocatalytic properties, the valence band (VB) energy of each sample was estimated from the valence band (VB) XPS measurements (Figure S10), considering aprevious study [83]. The results revealed VB maxima of 4.5 eV for the TaNbMCM-41 sample, very close to the VB potential of TaVMCM-41 (4.3 eV), 3.6 eV for TaCoMCM-41, and 4.4 eV for TaTiMCM-41. The conduction band (CB) potential of the synthesized materials was obtained as follows:

E_CB_ (vs. NHE) = E_VB_ (vs. NHE) − E_g_, (1)

where E_CB_ is the CB potential, E_VB_ is the VB potential, and E_g_ is the band gap energy [83,84].

**Figure 14:**
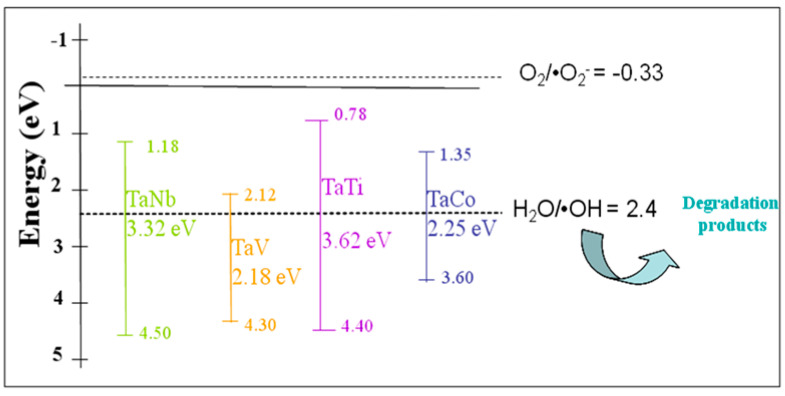
Energy band diagram for the bimetallic Ta/Me samples.

In the energy band diagram for the bimetallic Ta/Me samples (Figure 14) are represented the calculated and experimental values of the energy for the band gap (Table 3), conduction band (Equation (1)) and valence band (Figure S10), respectively. These values are compared with those of the necessary energy to generation of •O_2_^−^ and •OH oxidative radicals. It can be seen that the CB energy level is higher than the normal redox potentials of O_2_/•O_2_^−^ for all the bimetallic samples.

**Figure 15:**
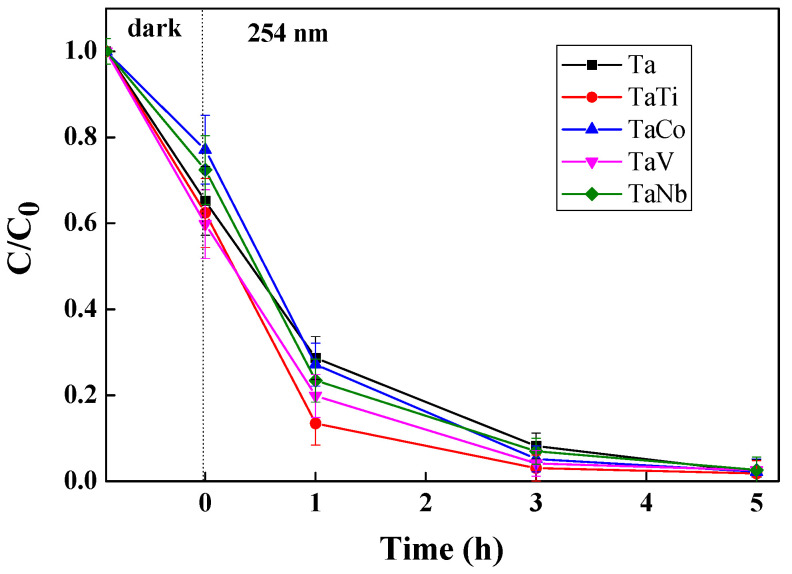
Photodegradation of methyl orange with H_2_O_2_ under UV light.

**Figure 16:**
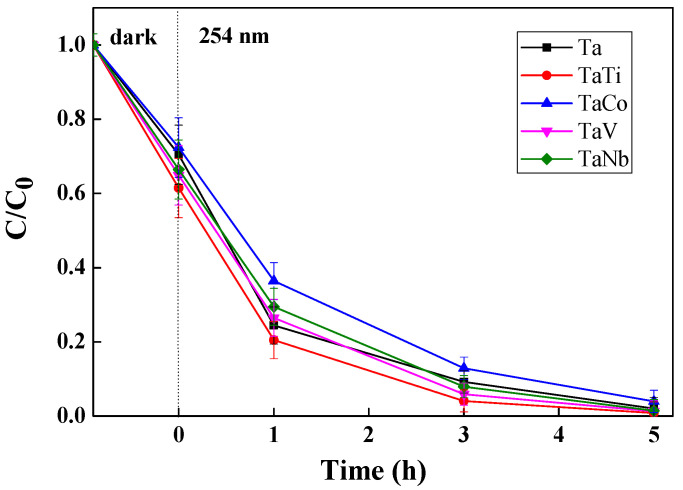
Oxidative photodegradation of phenol under UV light in thepresence of H_2_O_2_.

Since the VB energy value was more positive for all the samples than the H_2_O/•OH potential (+2.40 eV), •OH radicals were generated [80]. These radicals contributed to the degradation of the MO dye from the aqueous solution (Figure 14). Figure S11 shows a significant absorption of MO on the photocatalyst surface. However, under irradiation, the MO concentration decreases only in the case of the TaTi sample. The higher photocatalytic activity of the TaTi-MCM-41 sample could be due to the higher absorption (Figure 10) or its significant activation at 254 nm (Figure 11). The efficiency of the MO degradation increased significantly for all the photocatalysts (Figure 15) under the addition of H_2_O_2_. This is similar to the Fe_3_O_4_@SiO_2_@ZnO composite used for MO photodegradation under UV light [85]. We can consider that the high adsorption capacity is the effect of the hydroxyl groups from the photocatalyst surface. The FTIR [86] spectra indicated insignificant changes, compared to MCM-41, for similar materials (Nb-MCM-41, NbCo-MCM-41) obtained by direct synthesis. Thus, it was confirmed by the Raman data that there is no significant modification of the OH groups of silica by the immobilization of bimetals, except for small amounts of Me-OH bonds. Increased MO photodegradation efficiency was achieved by adding H_2_O_2_. As an electron acceptor, H_2_O_2_ generated hydroxyl radicals (H_2_O_2_+e^−^→OH^−^ +•OH). Thus, the possibility of photogenerated e^-^/h^+^ recombination was reduced, and the number of •OH radicals on the surface increased. Since the photogenerated electrons in the synthesized materials, except for the TaTi sample, cannot form •O_2_^−^ radicals with O_2_, the photocatalytic reactions are hindered by their recombination with the holes. This is the reasoning for the photocatalytic reactions performed in the presence of H_2_O_2_. Higher efficiency in phenol degradation was obtained for the TaTi and TaV samples (Figure 16).

The kinetics of the photodegradation reactions for methyl orange and phenol were described using the classical pseudo-first-order (PFO) and pseudo-second-order (PSO) models [87]. According to the correlation coefficient (R^2^) values, the PFO model fits better for all the catalysts except for the TaTi and TaV samples (Table 6). For these samples, the R^2^ values indicate PSO kinetics in MO photodegradation. This result can be explained by the Raman data (Figure 6), which indicate the existence of intense peaks attributed to the tetrahedral TO_4_ species (where T can be Si, Ti, or V).

The mechanism proposed for these oxidation reactions with hydrogen peroxide marked the formation of •OH with •O_2_^−^ species [81]. The above-presented results recommend the modified Ta-MCM-41 materials as catalysts for organic compound oxidation and confirm their possible application in photocatalytic reactions. The maximum degradation of MO and Ph was obtained for all the samples in the presence of H_2_O_2_ (Table 3). Due to the numerous factors affecting photocatalytic reactions, it is challenging to directly correlate band gap energy values with photodegradation efficiency, particularly in hybrid photodegradation processes. In addition, for the materials studied, the energy values of the valence bands cannot be compensated by the band gap energy, which can be seen in Figure S10. The degradation of these organic pollutants probably occurs due to a synergistic effect of adsorption and photocatalytic oxidation at the macro level. At the micro level, it is mainly due to the generation of numerous hydroxyl radicals, produced in the presence of H_2_O_2_ and through the photocatalytic process.

## 4. Conclusions

New bimetallic Ta/Ti, V, Co, and Nb mesoporous nanomaterials were synthesized using a direct method. The structure, texture, and morphology typical of the MCM-41 support were little influenced by the immobilization of the metals. The XPS results indicated low concentrations of metal species on the MCM-41 surface. This is obvious for the Ta and Nb samples for which the amount of metal is insignificant. These results are analogous to the published data about the low density of Vb metals, such as Ta and Nb, on the surface of the supporting silica framework. The second metal reduced the energy value of the band gap, but no significant effect on the photocatalytic properties was observed. The presence of Ta_2_O_5_ and partial (Nb, V) or complete (Ti) incorporation as (SiO)_3_Me=O into theMCM-41 framework were depicted by the Raman and UV–vis findings. Despite the resonant effect (namely UV–adsorption at the same wavelength as the Raman excitation), only Co_3_O_4_ was observed by Raman spectra deconvolution. The XPS highlighted, for the TaTi-MCM-41 sample, that framework titanium was the major component. The defects in the silica network, more significant for this sample, were also confirmed by XRD at a small angle. This sample also presented, both in the presence and in the absence of hydrogen peroxide, the highest photocatalytic activity.

All the catalysts were active in the oxidation with H_2_O_2_ of olefinic compounds (1,4-cyclohexadiene, cyclohexene, styrene). The second metal and slow addition of H_2_O_2_ favor the selectivity for epoxide by the oxidation of 1,4-cyclohexadiene and cyclohexene. The insignificant methyl orange photodegradation on all the catalysts was explained by a positive conduction band (CB) potential, higher than the normal redox potentials of O_2_/•O_2_^−^. In the presence of H_2_O_2_, methyl orange and phenol were completely photodegraded. As an electron acceptor, H_2_O_2_ captured photogenerated electrons, blocking their recombination with holes, and generated more hydroxyl radicals, increasing the photocatalytic activity.

## Figures and Tables

**Table 1:** The percentage of metals (Me) in the obtained samples ^a^.

Sample	Sample Code	Ta, %	Nb, %	V, %	Ti, %	Co, %	Ta/Me ^b^
Ta-MCM-41	Ta	5.36	-	-	-	-	-
TaNb-MCM-41	TaNb	5.36	3.28	-	-	-	0.829
TaV-MCM-41	TaV	5.55	-	1.96	-	-	0.789
TaTi-MCM-41	TaTi	5.34	-	-	4.23	-	0.329
TaCo-MCM-41	TaCo	3.33	-	-	-	2.44	0.445

^a^ Percentage weight from XRF; ^b^ Ta/Me molar ratio from XRF.

**Table 4:** Results of the catalytic tests.

Sample	1,4-Cyclohexadiene		Cyclohexene		Styrene	
	C (%)	S_HOL_ (%)	C (%)	S_HO_ (%)	C (%)	S_SO_ (%)
Ta	25.2	91.2	28.1	25.2	48.1	21.2
Ta Nb	81.1	85.2	65.7	71.2	76.4	65.0
TaV	36.1	84.5	32.4	78.9	89.0	66.8
TaTi	37.4	98.6	56,3	89.4	95.0	62.6
TaCo	64.0	89.0	64.2	84.4	78.2	71.2

**Table 5:** Summary of results for oxidation reactions catalyzed by Ta, Ti, V, Nb immobilized on silica supports.

Catalyst	Substrate	Reaction Conditions	C, %	S_EPO_, %	S_HOL_, %	S_DOL_/S_CO_, %	Ref.
Ta-SiO_2_	Styrene	104 mg, 6 h, 65 °C, H_2_O_2_, acetonitrile	37	36	-	-	[32]
Nb-KIT-6	Styrene	20 mg, 6 h, 50 °C, H_2_O_2_, methanol	51	13	-	-	[73]
NbV-MCM-41	Phenol	200 mg, 5 h, 80 °C, H_2_O_2_, H_2_O	2.55	-	100	-	[74]
Nb-MCM-41	Cys-cyclooctene	100 mg, 24 h, 80 °C, ethyl acetate	46	77	-	-	[75]
Ti-MCM-41	Styrene	20 mg, 36 h, 600 °C	48.8	72.3	-	27.7	[76]
Ti(OPr)_4_-MCM-41	Styrene	40 mg, 24 h, 70 °C, tert-butyl hydroperoxide, decane (acetonitrile)	68 (79)	44 (36)	-	-	[77]
Ta(OEt)_5_-MCM-41	Styrene	40 mg, 24 h, 70 °C, tert-butyl hydroperoxide, decane (acetonitrile)	59 (69)	24 (23)	-	-	[28]
Ta_2_O_5_·6SiO_2_	Cyclohexene	35 mg, 65 °C, 2 h, H_2_O_2_ acetonitrile	2.61	9.6	58	31.8	[17]
Ta-SBA-15	Cyclohexene	35 mg, 65 °C, 2 h, H_2_O_2_ acetonitrile	13.6	36	32.7	31.3	[17]

Abbreviations: Epoxide, EPO; Hydroxil, HOL; Diole, DOL; Cetone, CO.

**Table 6:** PFO and PSO rate constants for methyl orange dye and phenol degradation.

Sample	MO				Ph			
	PFO		PSO		PFO		PSO	
	k_1_ (min^−1^)	R^2^_adj_	k_2_ (Lmol^−1^min^−1^)	R^2^_adj_	k_1_ (min^−1^)	R^2^_adj_	k_2_ (Lmol^−1^min^−1^)	R^2^_adj_
Ta	0.67009 ± 0.01747	0.99729	40.13985 ± 11.16201	0.74893	0.66294 ± 0.03919	0.98617	5.51095 ± 1.60835	0.72864
TaTi	0.7217 ± 0.10731	0.91707	50.09469 ± 5.7599	0.94914	0.83548 ± 0.02653	0.99598	14.07729 ± 4.28598	0.70989
TaCo	0.694 ± 0.05896	0.97177	39.02368 ± 7.10775	0.87931	0.55547 ± 0.01693	0.99629	2.82933 ± 0.69338	0.79644
TaV	0.65621 ± 0.08449	0.93684	34.7876 ± 4.09714	0.94673	0.7582 ± 0.02175	0.99672	9.04504 ± 2.68858	0.72063
TaNb	0.64324 ± 0.04877	0.9774	32.06994 ± 6.38889	0.85814	0.72291 ± 0.03025	0.99303	7.68671 ± 2.43553	0.69138
Equation [87]	ln(C_0_/C) = k_1_t *		1/C-1/C_0_ = k_2_t *		ln(C_0_/C) = k_1_t *		1/C-1/C_0_ = k_2_t *	

* Notes: C_0_ and C are the Mo or Ph concentrations in the initial solution and after time t, and k_1_ and k_2_ are the rate constants for pseudo-first and -second order reactions, respectively.

**Table 2:** Variation of textural parameters.

Sample	Ta	TaNb	TaV	TaTi	TaCo
S_BET_ (m^2^/g)	867	856	862	826	817
V_BJH_ (cm^3^/g)	0.877	0.942	0.894	1.012	1.025
D_BJH_ (nm)	2.9	3.1	2.8	3.0	3.9

**Table 3:** Band gap energy (E_g_) of the samples and their photocatalytic efficiency in degradation of MO and Ph under UV light irradiation and the presence of H_2_O_2_.

		Ta	TaTi	TaNb	TaCo	TaV
**E_g_ (eV)**		5.64	3.62	3.32	2.25	2.18
MO Degradation Efficiency (%)	1 h	71.34	86.55	76.56	72.86	80.13
	3 h	91.79	96.92	93.01	94.78	95.85
	5 h	97.99	98.19	97.40	97.80	97.39
Ph Degradation Efficiency (%)	1 h	75.57	79.55	70.56	63.61	73.56
	3 h	90.79	95.92	92.10	87.12	94.10
	5 h	97.98	99.19	98.55	96.05	98.75

## Data Availability

The data presented in this study are available on request from the corresponding author.

## Supplementary Materials

The following supporting information can be downloaded at: https://www.mdpi.com/article/10.3390/nano14242025/s1. Figure S1: The high-angle XRD diffractograms of TaMe/MCM-41 samples; Figure S2: TEM images of Ta-MCM-41 (a); TaV-MCM-41 (b); TaTi-MCM-41 (c); and TaNb-MCM-41 and TaCo-MCM-41 samples; Figure S3: Raman spectra of the monometallic (Ta, V, Ti, Nb, Co)MCM-41 and MCM-41 samples; Figure S4: XPS full scan survey spectra for the bimetallic Ta/Me samples; Figure S5: XPS spectra of Ta4f for the bimetallic samples.; Figure S6: XPS spectra for Nb3d of TaNb-MCM-41 sample; Figure S7: XPS spectra for O1s of the bimetallic Ta/Me-MCM-41 samples; Figure S8. XPS Si2p high resolution spectra for the bimetallic Ta/Me-MCM-41 samples.Figure S9: Estimation of the band gap energy by the simplified analysis of the Tauc plot; Figure S10: XPS valence band spectra of the bimetallic Ta/Me samples. Linear fits and the valence band maximum energy are shown on each spectrum; Figure S11: Photodegradation of methyl orange in aqueous solution; Table S1: 2θ(100) position, interplanar spacing (d), and lattice parameter (a_0_) of the samples; Table S2: Peak position and assignments for the monometallic (Ta/Nb/Ti/Co/V)-MCM41 catalysts; Table S3: Peak position and assignments for the Ta and bimetallic Ta(Nb, T, Co, V) catalysts within 260–1200 cm^−1^ and 2600–4000 cm^−1^ ranges.
