# Supplementary material for: Bimetallic Mesoporous MCM-41 Nanoparticles with Ta/(Ti, V, Co, Nb) with Catalytic and Photocatalytic Properties
Source: Nanomaterials (Basel). 2024 Dec 16;14(24):2025. doi: 10.3390/nano14242025 (PMC11676168; doi:10.3390/nano14242025)
Supplement: Supplementary file 1 [file nanomaterials-14-02025-s001.zip › nanomaterials-3363546-supplementary.pdf]

Supplementary Data

Bimetallic Mesoporous MCM-41 Nanoparticles with Ta/(Ti, V, Co, Nb) with Catalytic and Photocatalytic Properties

Viorica Parvulescu, Gabriela Petcu, Nicoleta G. Apostol, Irina Atkinson, Simona Petrescu, Adriana Baran, Daniela C. Culita, Ramona Ene, Bogdan Trica and Elena M. Anghel

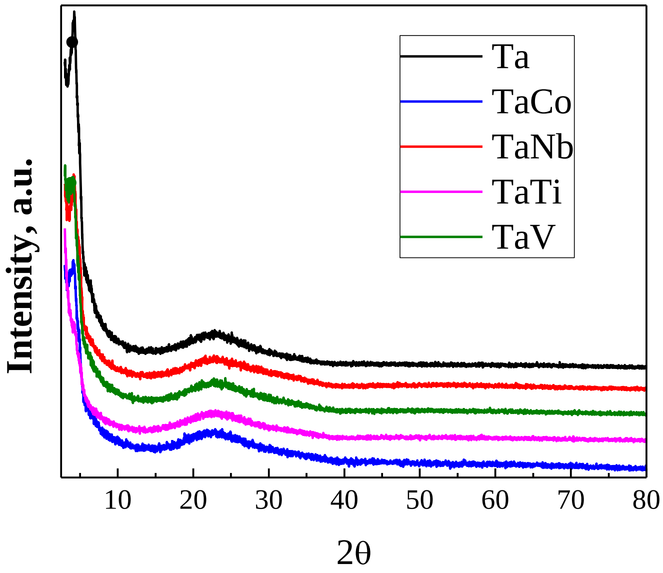

Figure S1. The high angles XRD diffractograms of TaMe/MCM-41 samples.

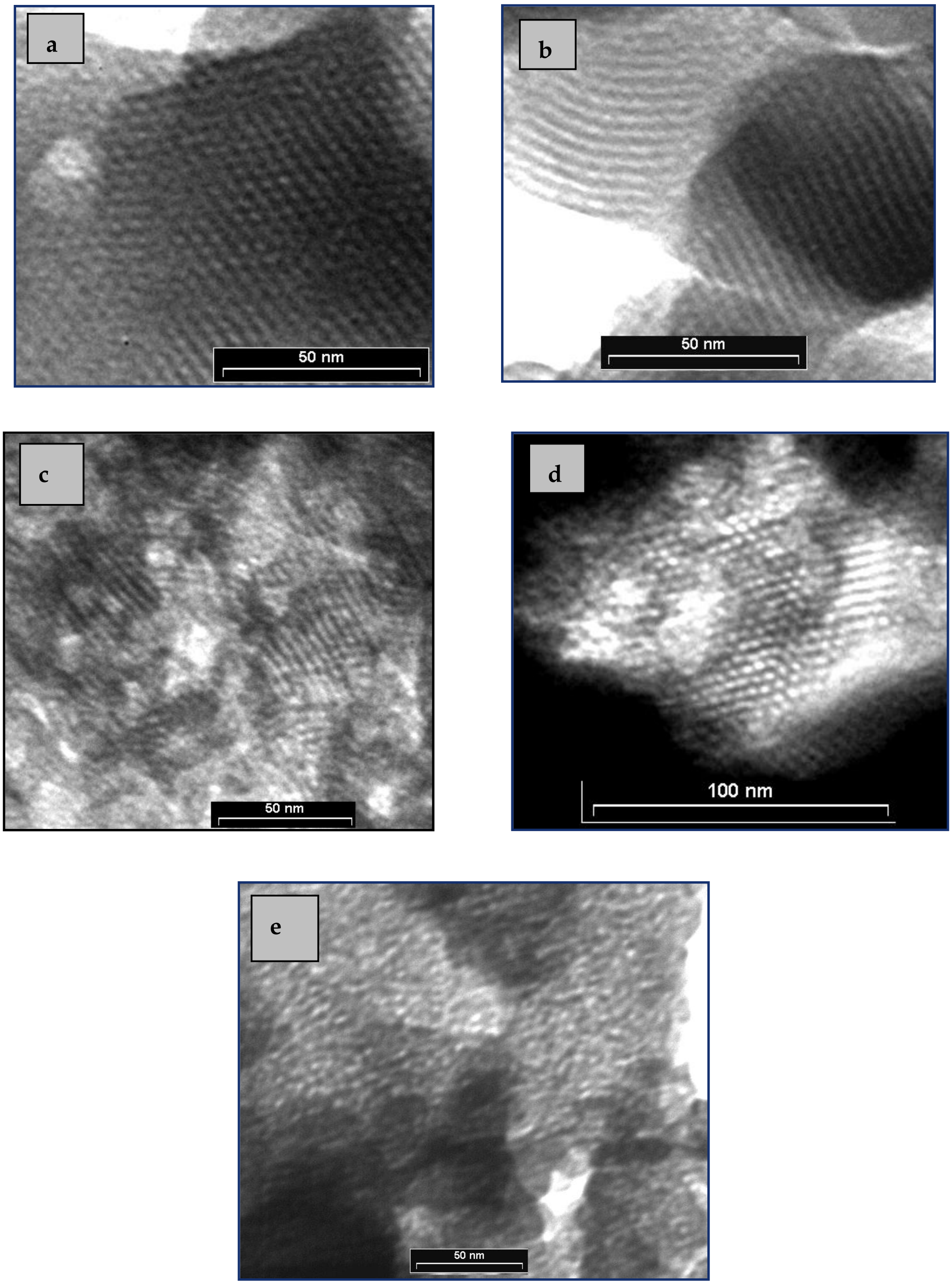

Figure S2. TEM images of Ta-MCM-41 (a); TaV-MCM-41 (b);
TaTi-MCM-41 (c); TaNb-MCM-41 (d) and TaCo-MCM-41 (e) samples.

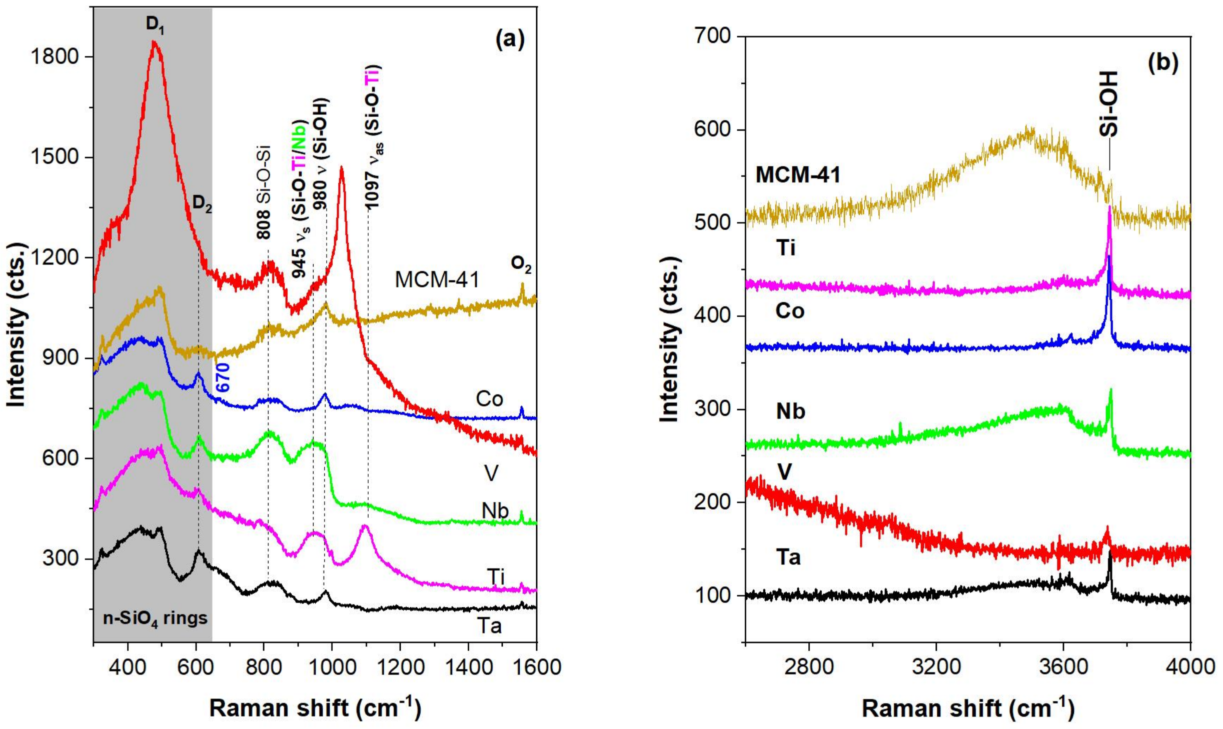

Figure S3. Raman spectra of the monometallic (Ta, V, Ti, Nb, Co)-MCM-41 and MCM-41 samples

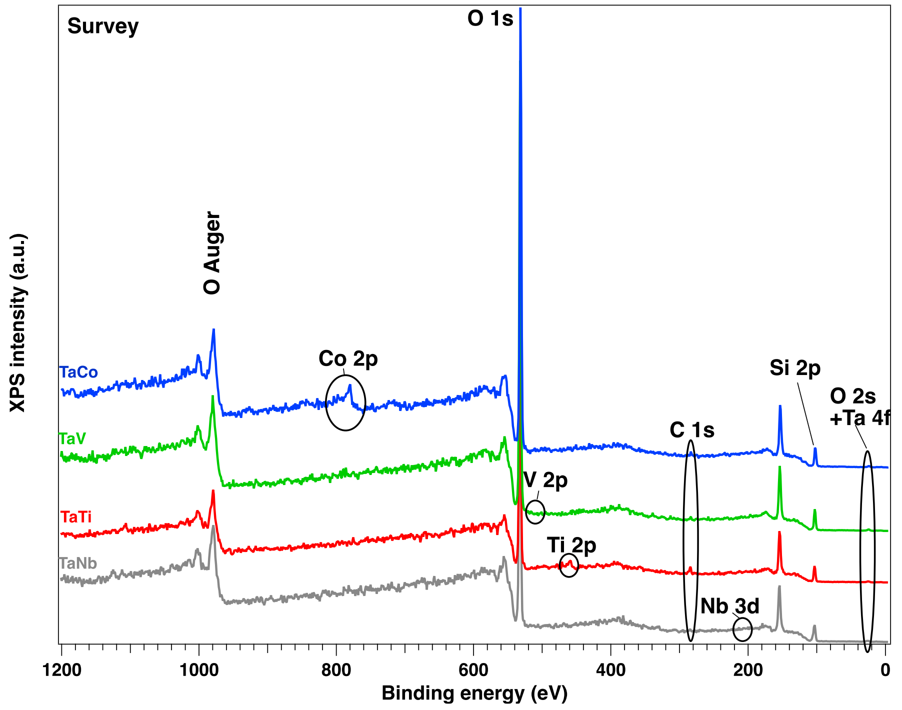

Figure S4. XPS full scan survey spectra for the bimetallic Ta/Me samples.

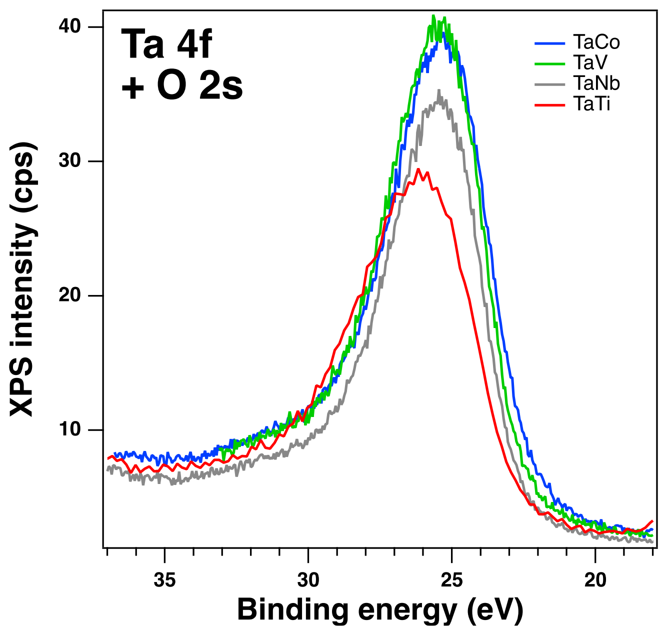

Figure S5. XPS spectra of Ta 4f for the bimetallic samples.

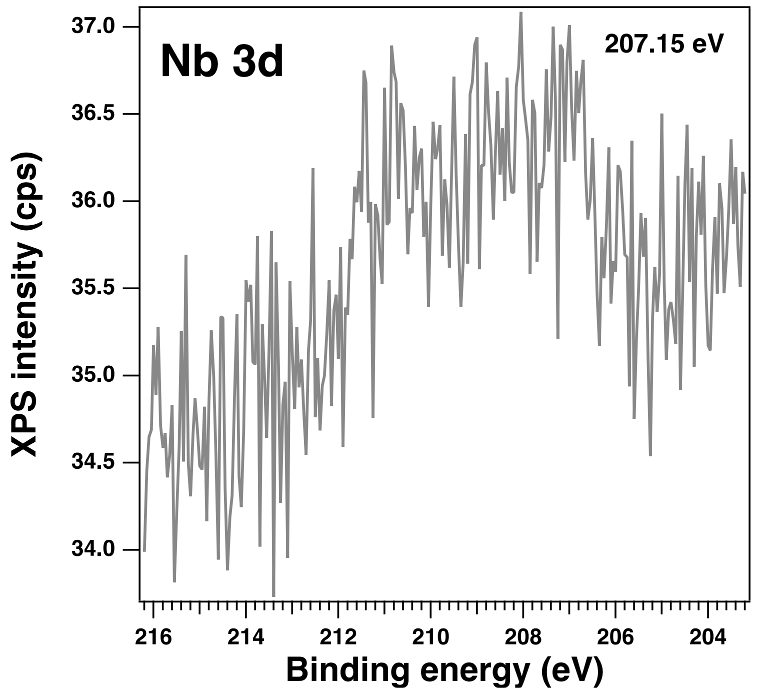

Figure S6. XPS spectra of Nb 3d for the sample TaNb-MCM-41 sample.

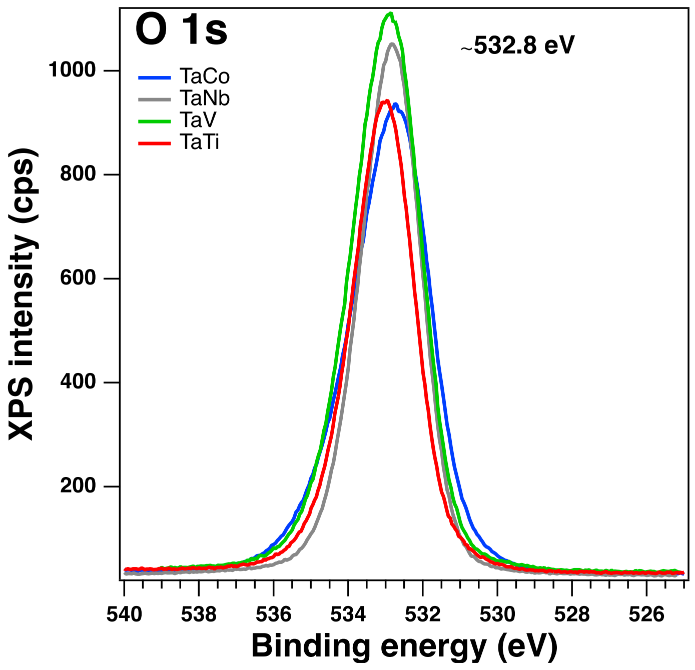

Figure S7. XPS O1s high resolution spectra for the bimetallic Ta/Me-MCM-41 samples.

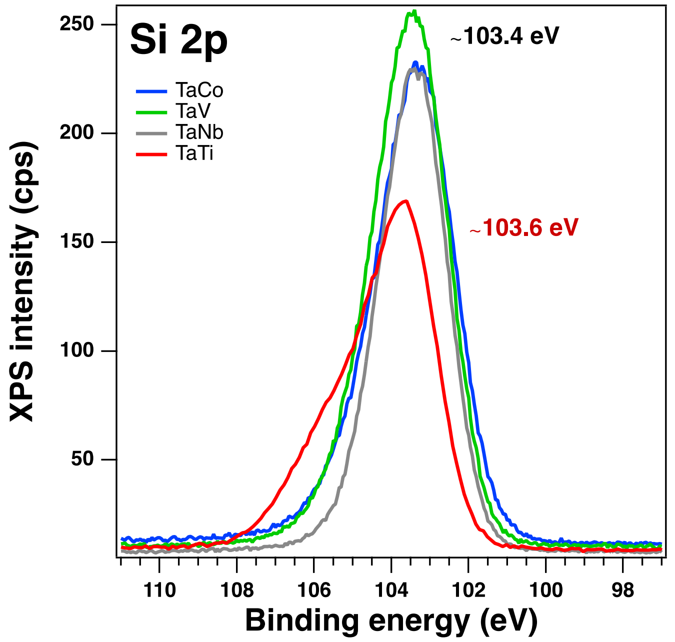

Figure S8. XPS Si2p high resolution spectra for the bimetallic Ta/Me-MCM-41 samples.

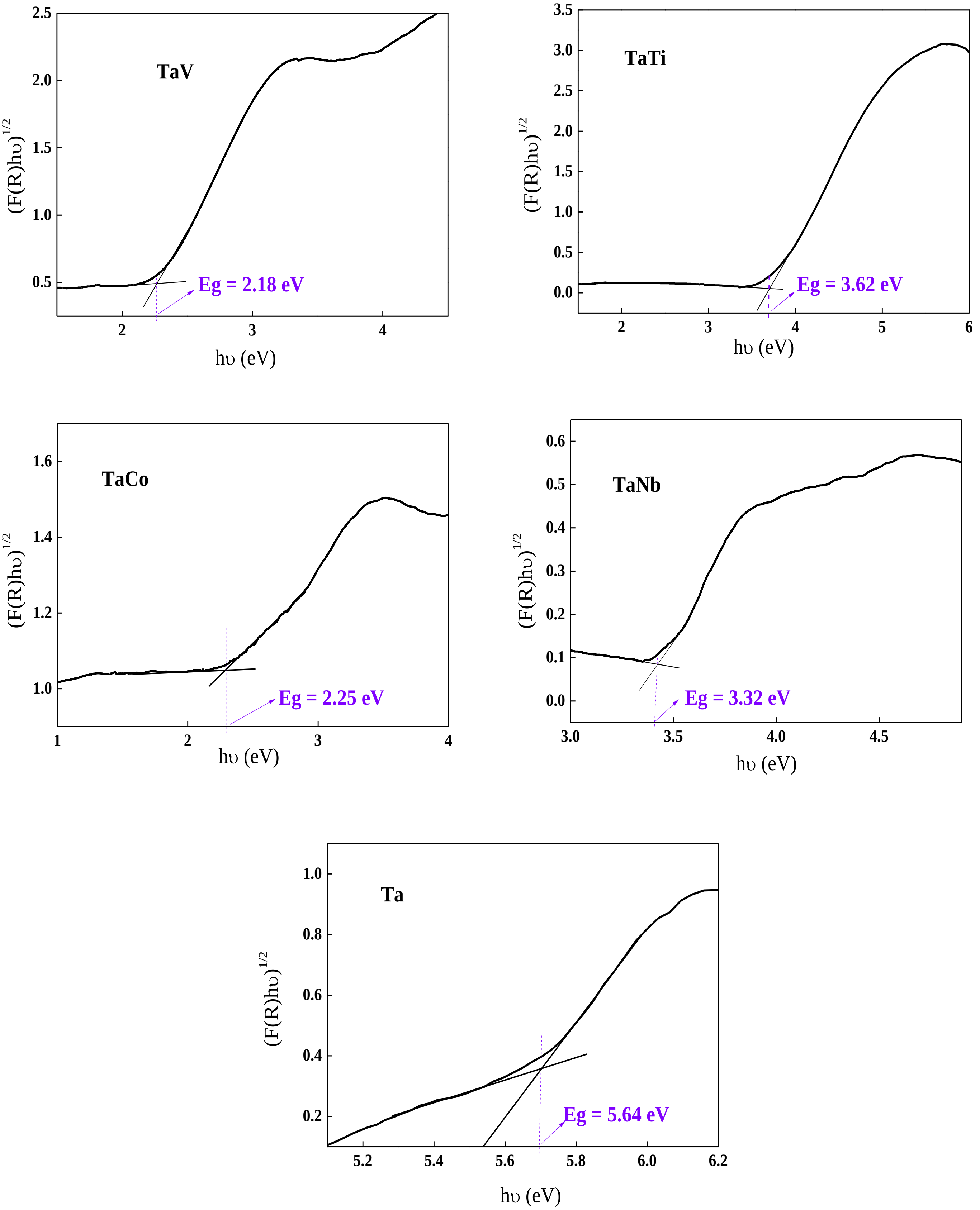

Figure S9. Estimation of the band gap energy by the simplified analysis of the Tauc plot.

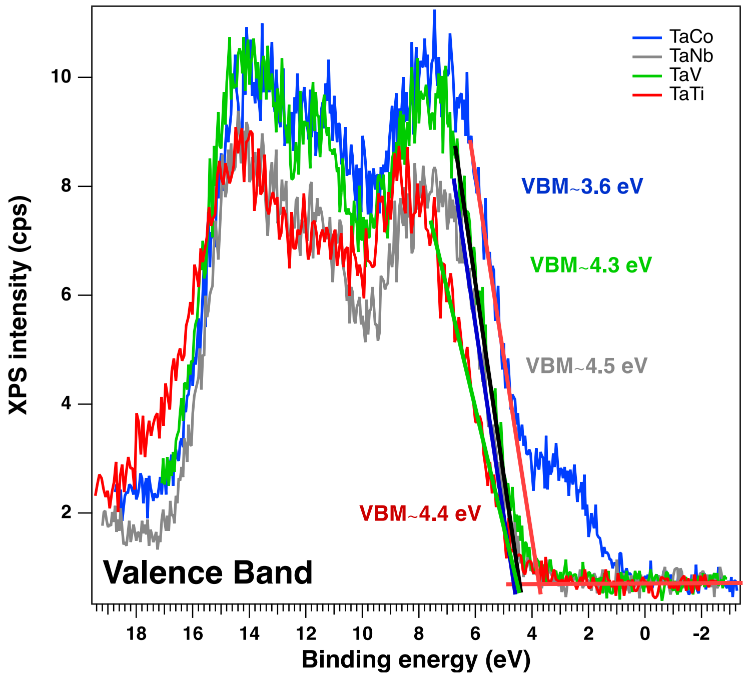

Figure S10. XPS valence band spectra of the bimetallic Ta/Me-MCM-41 samples. Linear fits and the valence band maximum energy are shown on each spectrum.

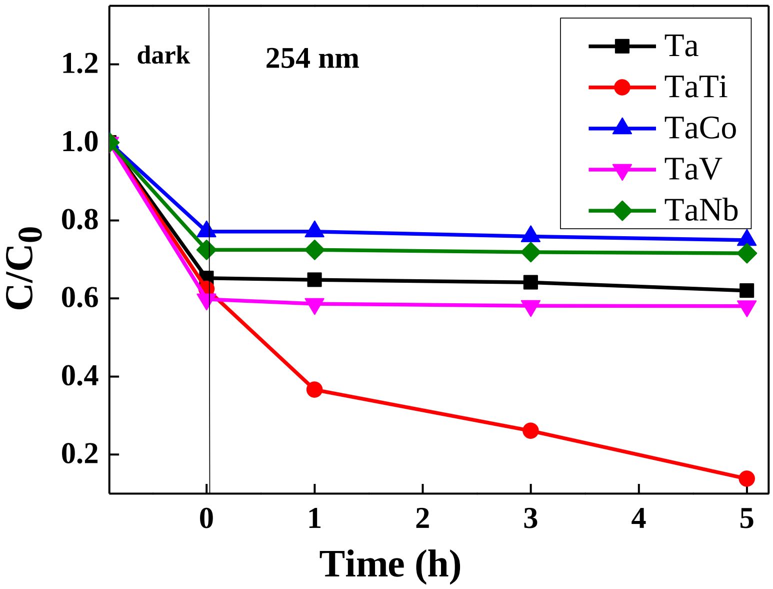

Figure S11. Photodegradation of methyl orange in aqueous solution.

Table S1. 2θ(100) position, interplanar spacing (d) and lattice parameter (a0) of the samples

Sample code	2θ $_{(100)}$ (deg)	$d_{(100)}$ (nm)	a_0 (nm)
**Ta**	2.31	3.82	4.42
**TaCo**	2.30	3.83	4.43
**TaTi**	2.40	3.67	4.24
**TaV**	2.33	3.77	4.36
**TaNb**	2.34	3.76	4.35

Table S2. Peak position and assignments for the monometallic (Ta/Nb/Ti/Co/V)-MCM41 catalysts.

**Peak position (cm^{-1})**					**Assignments**	**Reference**
**Ta**	**Nb**	**Ti**	**Co**	**V**	*<650 cm^{-1} n-SiO_4 rings*	42,43
				356	δ (O-V-O) in polymeric VO_x	44, 45
360	368				5,6,7-SiO_4 rings	42
441	443	440	436		5,6,7-SiO_4 rings	42
497	490	495	497	481	D_1 modes (4- SiO_4 rings)	42
				552	Silicalite framework breathing modes	44
608	610	607	608		D_2 modes (3- SiO_4 rings)	42
660					TaO_6	46
			664		CoO_6 in Co_3O_4	47
	822	800	798	821	$υ_s$ (Si-O-Si) and surface polymerized niobia	42, 46
828			838		$υ_s$ of the [SiO_4] units	42
	935				surface polymerized niobia species (820-935 cm^{-1})	46
958		954		958	$υ_s$ (Si-O-Si/Ti/V)	48,49
980	975		976		υ (Si-OH) and TaO_x species (965-980 cm^{-1})	42,46,50
				1028	Pyramidal structure of $(SiO)_3V{=}O$ when vanadium content is less than 2wt.% (1033-1036 cm^{-1})	44
1050	1091		1052		Antisymmetric stretching υ (Si-O)	42
		1100			$υ_{as}$ (Si-O-Ti) with Ti^{4+}	48
1185	1150			1123		
0.9923	0.9918	0.9847	0.9897		R^2	
					>3000 cm^{-1}	50
	3233				ν(OH) moodes of H_2O involved in a tetrahedral structure	50
3600	3594	3595	3623		MeO-H and free H_2O	49,50
3700	3690		3695		HO-H dangling	50
3747	3746	3743	3745	3740	SiO-H and Free SiO-H	50

Table S3. Peak position and assignments for the Ta and bimetallic Ta(Nb, T, Co, V) catalysts within 260-1200 cm^{-1} and 2600-4000 cm^{-1} ranges.

Peak position (cm^{-1})					Assignments	Ref.
**Ta**	**Ta Nb**	**TaTi**	**TaCo**	**TaV**	***<650 cm^{-1} n-SiO_4 rings***	42,43
				355	δ (O-V-O)	44,45
360	348	375	362		5,6,7-SiO_4 rings	42
441	447		435		5,6,7-SiO_4 rings and E_g modes of the extra-framework rutile (445 cm^{-1})	42
497	496	497	495	486	D_1 modes (4- SiO_4 rings), bending modes of the framework Me/Si-O-Si speciation	42
608	608	607	612	609	D_2 modes (3- SiO_4 rings)	42
	615	616			υ(Nb-O-Nb) polymerized Nb species (607-650 cm^{-1}) and A_{1g} modes in extra-framework rutile (612 cm^{-1})	53 and 43
660		685	670		υ(Ta-O) in TaO_6	46
	689				Nb_2O_5	53
			707		Co_3O_4 (690 cm^{-1})	47
	801	798	796		$υ_s$ modes of the siloxane bridges Si-O-Si	42
828			830		$ν_s$ modes of the siloxane bridges Si-O-Si	42
958		945			υs (Si-O-Ti/Nb)	49, 51
980	978	985	973		υ (Si-OH), ν(Si-NBO) in Q^2 units, υ(Nb=O) of isolated NbO_4 and TaO_x species (965-980 cm^{-1})	42,52-55 and 18
				1030	$(SiO)_3V=O$ stretching modes	26
				1060	Shorter V=O bonds	56
1050	1056		1065		Q^4 units in silica framework	42,52
		1097			$υ_{as}$ (Si-O-Ti) with Ti^{4+}	48
1185		1164	1187			
					>3500 cm^{-1} (hydroxyl stretching modes)	
3617		3602	3623	3547	Me-OH and free H_2O	49
3746	3744	3744	3744	3738	Isolated Si-OH in MCM-41	50
0.9923	0.9943	0.9949	0.9857	0.9970	R^2	

νs,as-symmetric, asymmetric stretching, δ-bending vibrations. NBO is non-bridging oxygen. Q2 represents SiO_4 tetrahedra with 2NBO.
